# Cardiovascular adverse events in patients with lung cancer treated with immune checkpoint inhibitors: a nationwide database study

**DOI:** 10.1093/oncolo/oyaf151

**Published:** 2025-06-23

**Authors:** Tsuyoshi Isawa, Shintaro Togashi, Masataka Taguri, Yukihiro Toi, Shunichi Sugawara, Shigeru Toyoda

**Affiliations:** Department of Cardiology, Sendai Kousei Hospital, Sendai 981-0914, Japan; Department of Nursing Care, Sendai Kousei Hospital, Sendai 981-0914, Japan; Center for Outcomes Research and Economic Evaluation for Health, National Institute of Public Health, Wako 351-0197, Japan; Department of Health Data Science, Tokyo Medical University, Tokyo 160-8402, Japan; Department of Pulmonary Medicine, Sendai Kousei Hospital, Sendai 981-0914 , Japan; Department of Pulmonary Medicine, Sendai Kousei Hospital, Sendai 981-0914 , Japan; Department of Cardiovascular Medicine, Dokkyo Medical University, Mibu 321-0293, Japan

**Keywords:** cardiotoxicity, cardio-oncology, lung neoplasms, database study

## Abstract

**Background:**

Large, diverse cohort studies are essential for determining the incidence and risk factors of major adverse cardiovascular events (MACEs) associated with immune checkpoint inhibitors (ICIs). This study aimed to (1) compare the incidence of MACEs in primary lung cancer patients receiving ICIs versus those receiving non-ICI chemotherapy, and (2) identify risk factors for MACEs in ICI-treated patients.

**Materials and methods:**

We performed a retrospective analysis of primary lung cancer patients using a nationwide Japanese database. Patients were stratified by their use of ICIs, and after propensity score matching, outcomes were evaluated over 180 days.

**Results:**

The study included 743 propensity-matched patients in each cohort. The median follow-up period was 329 days (interquartile range, 147-625). At 180 days, 4.0% of ICI-treated patients experienced MACEs, significantly higher than those treated with non-ICI chemotherapy (hazard ratio [HR], 1.98; 95% confidence interval [CI], 1.07-3.69; *P*-value = .030). Among MACEs, myocarditis and pericarditis occurred significantly more frequently in patients receiving ICI treatment than in those receiving non-ICI chemotherapy (*P*-value = .048 for both outcomes). No significant differences were observed in other MACE components. In multivariable analysis, chronic renal failure (HR, 2.16; 95% CI, 1.05-4.46; *P*-value = 0.038) and prior heart failure (HR, 3.08; 95% CI, 1.86-5.11; *P*-value < 0.001) were significant risk factors for MACEs.

**Conclusion:**

ICI treatment was associated with more frequent MACEs, primarily due to myocarditis and pericarditis. Additionally, prior heart failure and chronic renal failure were key risk factors for MACEs.

**Clinical trial registration:**

UMIN000051698

## Graphical Abstract

**Graphical Abstract:**
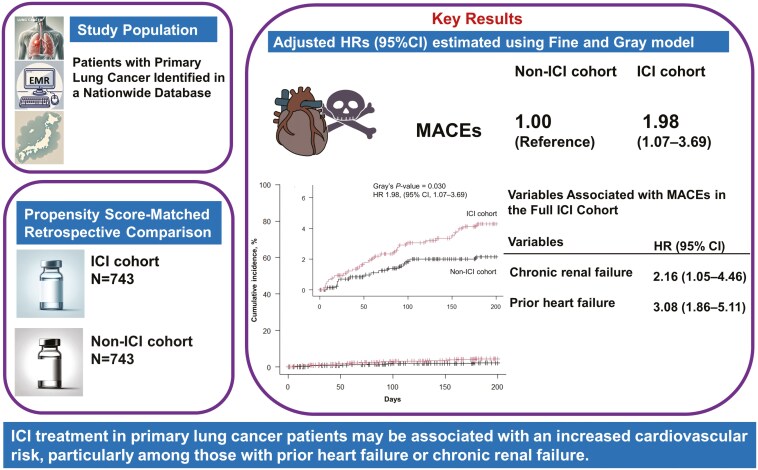

**Implications for practice**

This study, one of the largest real-world data analyses to date, demonstrated a significant increase in cardiovascular events among Japanese lung cancer patients treated with immune checkpoint inhibitors (ICIs) compared to those receiving non-ICI chemotherapy. Furthermore, it identified that cardiovascular events in ICI-treated patients were significantly associated with prior heart failure and concurrent chronic renal failure. The observed increase in cardiovascular events among lung cancer patients with prior heart failure or chronic renal failure receiving ICI treatment highlights the need for larger studies focusing on this high-risk subgroup to better evaluate the balance between therapeutic benefits and cardiovascular risks.

## Introduction

Immune checkpoint inhibitors (ICIs) are associated with clinically significant cardiovascular adverse events.^1^ Although ICI-related myocarditis has garnered considerable attention in the literature, emerging evidence underscores a broader spectrum of ICI-related cardiovascular adverse events. The recently published clinical practice guidelines from the American Society of Clinical Oncology categorize the events occurring during or after ICI treatment as cardiovascular immune-related adverse events, encompassing not only myocarditis but also pericarditis, arrhythmias, and heart failure.^2^ In addition, acute coronary syndromes have recently been identified as a manifestation of cardiovascular adverse events.^3^ Most existing pieces of evidence indicating an increased risk of cardiovascular adverse events originate from observational studies and clinical trials, reporting incidence rates of 0.6-%-13.3%.^4–7^ However, owing to limited sample sizes, uncommon adverse events are difficult to detect in single- or two-center observational studies. Furthermore, in cancer-related clinical trials, cardiovascular adverse events may be underreported because patients with cardiovascular comorbidities or renal failure are frequently excluded from these studies.^8,9^ Indeed, cardiovascular adverse events have been reported in only 0.6% of patients enrolled in clinical trials.^7^ Thus, to accurately assess cardiovascular risk in real-world settings, larger and more diverse cohort studies are essential. In this context, nationwide database studies provide unparalleled scale and generalizability. Moreover, considering their distinct cardiovascular risk profiles and higher inherent susceptibility to cardiovascular events, Asian populations require targeted investigation.^10^ Although studies on Western populations have explored ICI-related cardiovascular adverse events,^11,12^ few such studies have focused on Asian populations. This study aimed to report the incidence of and potential risk factors for cardiovascular adverse events in patients with primary lung cancer treated with ICIs, utilizing a nationwide database from Japan.

## Materials and methods

### Data source

This population-based retrospective observational cohort study utilized the RWD database administered by the Health, Clinic, and Education Information Evaluation Institute (Kyoto, Japan), with support from Real World Data, Co., Ltd.^13^ Electronic medical records, claims, and Diagnosis Procedure Combination data are included in the database.^14^ As of November 2022, the database encompassed information from 23 million patients across 229 medical institutions nationwide, encompassing approximately 1.3% of the Japanese population, and contained data on patient baseline characteristics, diagnoses, diseases (eg, International Classification of Diseases, Tenth Revision [ICD-10] codes), cancer stage classifications, treatment modalities, and laboratory results including serum creatinine and cardiac troponins (troponin T, troponin I, or qualitative tests for both). The investigators had restricted access to the database population, with data provided through an anonymized dataset managed by a third-party data custodian. Access was granted only to specific study parameters under Institutional Review Board-approved protocols. This study followed the RECORD statement guidelines for reporting observational studies using routinely collected health data (**Supplementary Table S1**). This study was registered at the University Hospital Medical Information Network Clinical Trials Registry, as accepted by the International Committee of Medical Journal Editors (No. UMIN000051698) and was approved by the Institutional Review Board of Sendai Kousei Hospital (approval number, 3-46; approval date, August 25, 2021). As this study was a retrospective analysis of existing data, written informed consent was waived.

### Patient selection and cohort entry date definition

The database, containing patient-related data from May 1, 2016 to April 30, 2021, was examined. Patients with primary lung cancer were identified using ICD-10 codes (C34.0, C34.1, C34.2, C34.3, or C34.9), Japan-specific medical procedure codes, and individual drug codes unique to the National Health Insurance drug price standard used in insurance claims in Japan (YJ codes). The accuracy of identifying patients with primary lung cancer in this database was very high, with approximately 99% of patients assigned a relevant diagnostic code having confirmed disease.^15^ The following patients were excluded from this study: (1) the following patients were excluded from this study: (1) those with secondary pulmonary tumors originating from non-pulmonary primary cancers (ICD-10 code C78.0); (2) those without a minimum look-back period of 180 days before cohort entry; (3) those with prior use of anthracyclines with high cardiotoxicity potential, including doxorubicin, daunorubicin, epirubicin, idarubicin, pirarubicin, and amrubicin, during the look-back period; and (4) those with a history of specific malignancies, including multiple myeloma, chronic myelogenous leukemia, cervical cancer, ovarian cancer, hepatocellular carcinoma, gastric cancer, rectal cancer, colon cancer, malignant lymphoma, breast cancer, and urothelial cancer, for which highly cardiotoxic antimalignancy drugs, such as proteasome inhibitors, tyrosine kinase inhibitors, vascular endothelial growth factor inhibitors, rituximab, and anthracycline antitumor agents, are widely employed in clinical practice.

The ICI cohort comprised patients with primary lung cancer who received ICI treatment for the first time during the study period (**Supplementary Table S2**). For the ICI cohort, the cohort entry date (index date) was defined as the date of the first ICI administration recorded in the database. No records of prior ICI use within 180 days preceding the cohort entry date were noted. The ICI cohort also included patients who underwent dual ICI therapy combined with chemotherapy. Notably, the ICI cohort included patients with a history of specific non-ICI anticancer drug use within the 180-day look-back period when they received any ICIs for the first time in the database, indicating that those who received ICIs as second-line or later treatments were included in the ICI cohort. The non-ICI cohort comprised patients with primary lung cancer who received specific non-ICI chemotherapy for the first time and who had not received any ICI treatment during the 180-day look-back period or within 180 days following the cohort entry date. For the non-ICI cohort, the cohort entry date (index date) was defined as the date of the first prescription of the specific non-ICI anticancer drugs recorded in the database. The incidence of major adverse cardiovascular events (MACEs) was assessed during a predefined 180-day period following cohort entry. Patients were then followed until censoring, which occurred at death, loss to follow-up, or the end of the observation period. The incidence of MACEs and individual components, including myocarditis, acute coronary syndromes, clinically relevant arrhythmias, heart failure, cardiac or sudden death, and pericarditis, was compared between the ICI and non-ICI cohorts. The study design diagram is presented in **Supplementary Figure S1**.

### The definitions of comorbidity and concomitant pharmacotherapy

Comorbidities were defined as the presence of the ICD-10 code and the Japan-specific medical procedure code and/or the YJ code within the 180-day look-back period before the cohort entry date. Concomitant pharmacotherapy was defined as the administration of specific drugs according to the YJ code within the 180-day look-back period before the cohort entry date. The definitions of comorbidities and concomitant pharmacotherapy are provided in **Supplementary Table S2**.

### The definition of outcomes

A composite of MACEs at 180 days, comprising one or more of the following events: myocarditis; acute coronary syndromes, including myocardial infarction, unstable angina pectoris, and vasospastic angina; clinically relevant arrhythmias; heart failure; cardiac or sudden death; and pericarditis, was the primary outcome. The individual components of the composite outcome analyzed separately comprised the secondary outcomes. Outcomes were defined using separate algorithms (**Supplementary Table S3**) based on a combination of ≥2 variables, including diagnoses, treatments, procedures, and laboratory test results.

### Statistical analysis

Continuous and categorical variables were presented as medians (interquartile ranges [IQR]) and frequencies (percentages), respectively. Patients were matched in a 1:1 ratio using propensity score matching with the nearest neighbor method, applying a caliper width equal to 0.2 of the standard deviation of the propensity score logit. Propensity scores were calculated using logistic regression, incorporating variables including age, sex, body mass index (BMI), chronic renal failure, prior myocardial infarction, and prior heart failure. These variables were selected on the basis of clinical expertise. Smoking status and cancer stage were excluded as covariates in the propensity score matching model for the following reasons: (1) both had high proportions of missing data (18.5% and 22.0%, respectively), and imputing binary (smoking) and categorical (stage) variables raises accuracy concerns; and (2) we assumed that adjusting for established smoking-related conditions such as prior myocardial infarction and heart failure would partially account for smoking-related cardiovascular risk. Negligible differences between cohorts in the measured variables were indicated by an absolute standardized mean difference of 0.10 or less. Gray’s test was employed for comparing the cumulative incidence of outcomes with a competing risk (all-cause death or noncardiac death/nonsudden death, as appropriate) between the two cohorts (matched ICI vs. matched non-ICI cohorts). A univariable Fine and Gray competing risk model was used to evaluate the risk of MACEs and individual outcomes, presenting hazard ratios (HRs) and their corresponding 95% confidence intervals (CIs) in the comparison between the two cohorts. All-cause death was considered a competing event for myocarditis, acute coronary syndromes, clinically relevant arrhythmias, heart failure, and pericarditis, while noncardiac death/nonsudden death was a competing event for MACEs and cardiac or sudden death. For all time-to-event analyses, patients were followed from their cohort entry date to the first occurrence of each outcome event, loss of fee-for-service inpatient or outpatient coverage, death, or June 1, 2021.

To identify potential risk factors for MACEs and individual components in the full ICI cohort, the multivariable Fine and Gray competing risk model was employed instead of the Cox proportional hazards model to evaluate the association between ICI treatment and the incidence of MACEs or their individual components, as it better accounts for competing events, yielding a more precise risk estimate under such conditions. Potential risk factors for MACEs were initially screened using a univariable Fine and Gray regression model, with a *P-*value of < .25 considered statistically significant for univariable screening regressions. Subsequently, to estimate HRs and 95% CIs, a multivariable Fine and Gray regression model integrating variables with a *P-*value of < 0.25 from the univariable analysis was constructed. A subgroup analysis was conducted to evaluate the risk of ICI plus platinum-based chemotherapy (carboplatin or cisplatin) versus non-ICI chemotherapy following propensity score matching for patient backgrounds.

All statistical analyses were performed using EZR software (version 1.53; Saitama Medical Center, Jichi Medical University; http://www.jichi.ac.jp/saitama-sct/SaitamaHP.files/statmedEN.html), a graphical user interface for R (The R Foundation for Statistical Computing, Vienna, Austria), with a two-sided *P*-value of < .05 indicating statistical significance. The primary and secondary outcomes underwent a complete case analysis. As part of the sensitivity analysis, multiple imputation was performed to address missing values in the binary covariate smoking status and the categorical covariate cancer stage. After imputation, both smoking status and cancer stage were added to the set of covariates used for propensity score matching in the main analysis to evaluate the robustness of the results. Missing data were addressed using the multiple imputation by chained equation approach, implemented with the IterativeImputer from the Scikit-learn library, with 20 iterations. All imputations assumed that the missing data were missing at random. Moreover, using a multivariable Fine and Gray competing risk model, further sensitivity analysis was performed to estimate the HR for the primary outcome (MACEs) in the full study cohort.

## Results

### Baseline patient characteristics and unmatched cohort analysis

**Figure 1.**
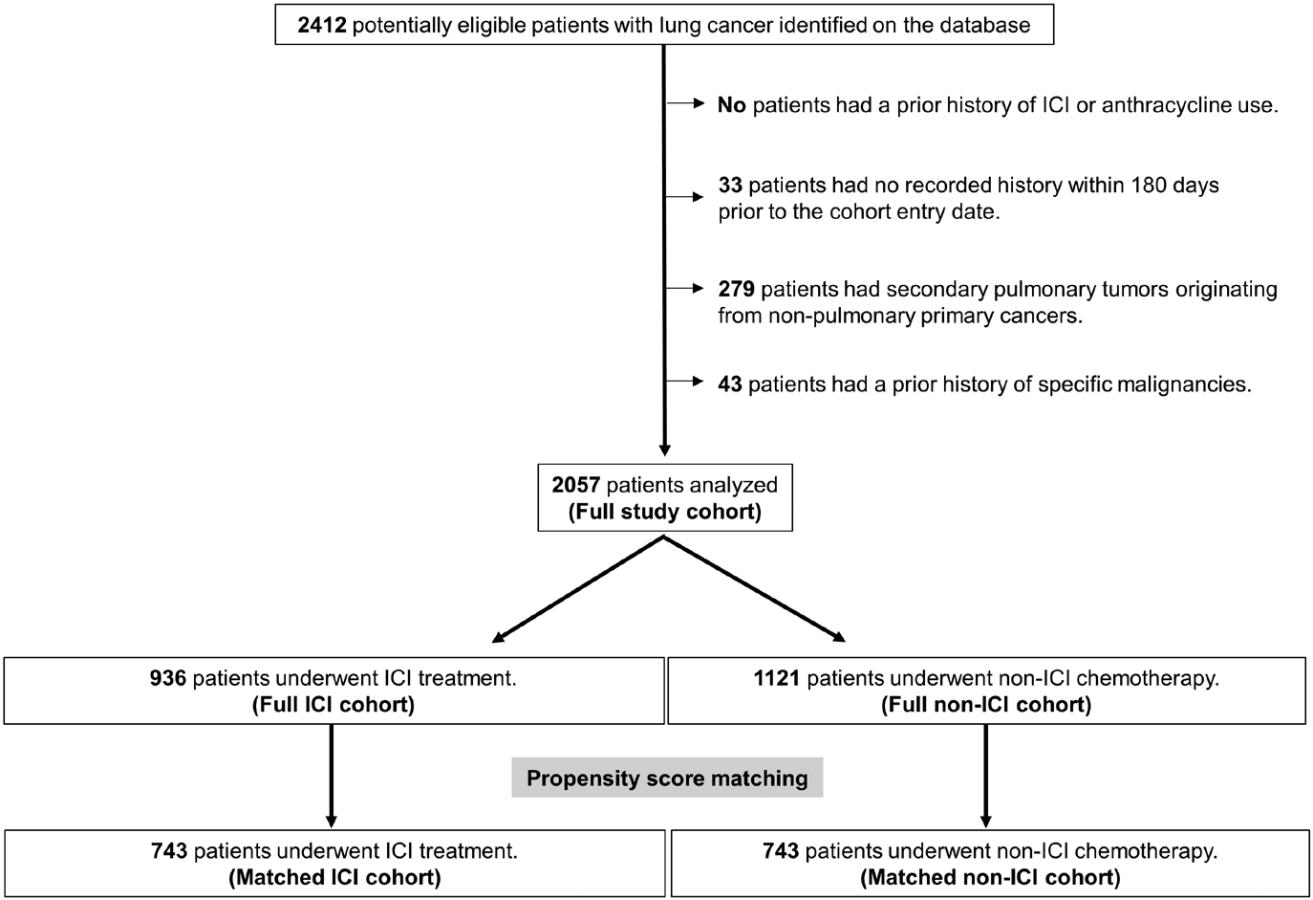
Study flow. In total, 2421 potentially eligible patients with lung cancer were identified on the database. Of these patients, 936 underwent ICI treatment and 1121 underwent non-ICI chemotherapy. Abbreviation: ICI, immune checkpoint inhibitor.

A total of 10,210 patients with primary lung cancer were extracted during the study period, identified solely based on ICD-10 codes (C34.0, C34.1, C34.2, C34.3, and C34.9). Among those, 2,412 potentially eligible patients diagnosed with lung cancer were identified in the database from May 1, 2016 to April 30, 2021. After applying the exclusion criteria, the final cohort (full study cohort) comprised 2,057 patients with primary lung cancer who met the following criteria: (1) assigned ICD-10 codes C34.0, C34.1, C34.2, C34.3, or C34.9; and (2) availability of baseline characteristics and treatment information. Of these 2057 patients, all covariates were complete except for BMI, smoking status, and cancer stage, which were missing in 13.8%, 18.5%, and 22.0% of cases, respectively. The full ICI cohort included 936 patients who received ICI treatment, whereas the full non-ICI cohort encompassed 1121 patients who received non-ICI chemotherapy. After matching, 743 patients remained in the matched ICI and matched non-ICI cohorts (**Figure 1**). The baseline patient characteristics are presented in Table 1**and****Supplementary Figure S2**, indicating that the matched ICI and matched non-ICI cohorts are well balanced with respect to baseline characteristics. Furthermore, details of the ICI treatment regimens are shown in **Supplementary Table S4**. Regarding ICI treatment characteristics, the prematched and postmatched ICI cohorts were considered balanced. Although the full ICI cohort showed higher HR for MACEs than the full non-ICI cohort, the difference in MACEs was not statistically significant (HR, 1.53; 95% CI, 0.92-2.52; *P*-value = .099) (**Supplementary Table S5**).

**Table 1.** Baseline characteristics of patients before and after propensity-score matching.

Variables	Before propensity-score matching			After propensity-score matching ^a^^)^		
	Full ICI cohort (*n* = 936)	Full non-ICI cohort (*n* = 1121)	SMD ^b^^)^	Matched ICI cohort (*n* = 743)	Matched non-ICI cohort (*n* = 743)	SMD ^b^^)^
Age, median (IQR), years	71.0 (66.0-76.0)	73.0 (68.0-79.0)	0.23	72.0 (67.0-77.0)	72.0 (67.0-77.0)	0.03
Sex (male), *n* (%)	692 (73.9)	232 (62.8)	0.24	530 (71.3)	537 (72.3)	0.02
Body mass index, median (IQR), kg/m^2^	21.8 (19.4-24.2)	22.1 (20.0-24.3)	0.09	22.0 (19.50-24.4)	22.0 (19.9-24.1)	0.02
Diabetes, *n* (%)	146 (15.6)	140 (12.5)	0.09	118 (15.9)	99 (13.3)	0.07
Hypertension, *n* (%)	103 (11.0)	153 (13.7)	0.08	85 (11.4)	90 (12.1)	0.02
Dyslipidemia, *n* (%)	25 (2.7)	50 (4.5)	0.10	24 (3.2)	33 (4.4)	0.06
Chronic renal failure, *n* (%)	84 (9.0)	52 (4.6)	0.17	55 (7.4)	45 (6.1)	0.05
Prior myocardial infarction, *n* (%)	22 (2.4)	33 (2.9)	0.04	18 (2.4)	16 (2.2)	0.02
Prior heart failure, *n* (%)	164 (17.5)	217 (19.4)	0.05	138 (18.6)	147 (19.8)	0.03
Chronic obstructive pulmonary disease, *n* (%)	110 (11.8)	94 (8.4)	0.11	88 (11.8)	71 (9.6)	0.07
Chronic liver disease, *n* (%)	271 (29.0)	318 (28.4)	0.01	222 (29.9)	209 (28.1)	0.04

Abbreviations: ICI, immune checkpoint inhibitor; IQR, interquartile range; SMD, standardized mean difference.

^a)^Patients were matched on the basis of a 1:1 ratio, using a propensity score-based procedure involving age, sex, body mass index, prior myocardial infarction, prior heart failure, and chronic renal failure. The caliper used for matching was set at 0.2.

^b)^An absolute SMD of ≤0.10 indicates a negligible difference in the measured variables between the cohorts.

### Matched cohort, sensitivity, and subgroup analyses

**Figure 2.**
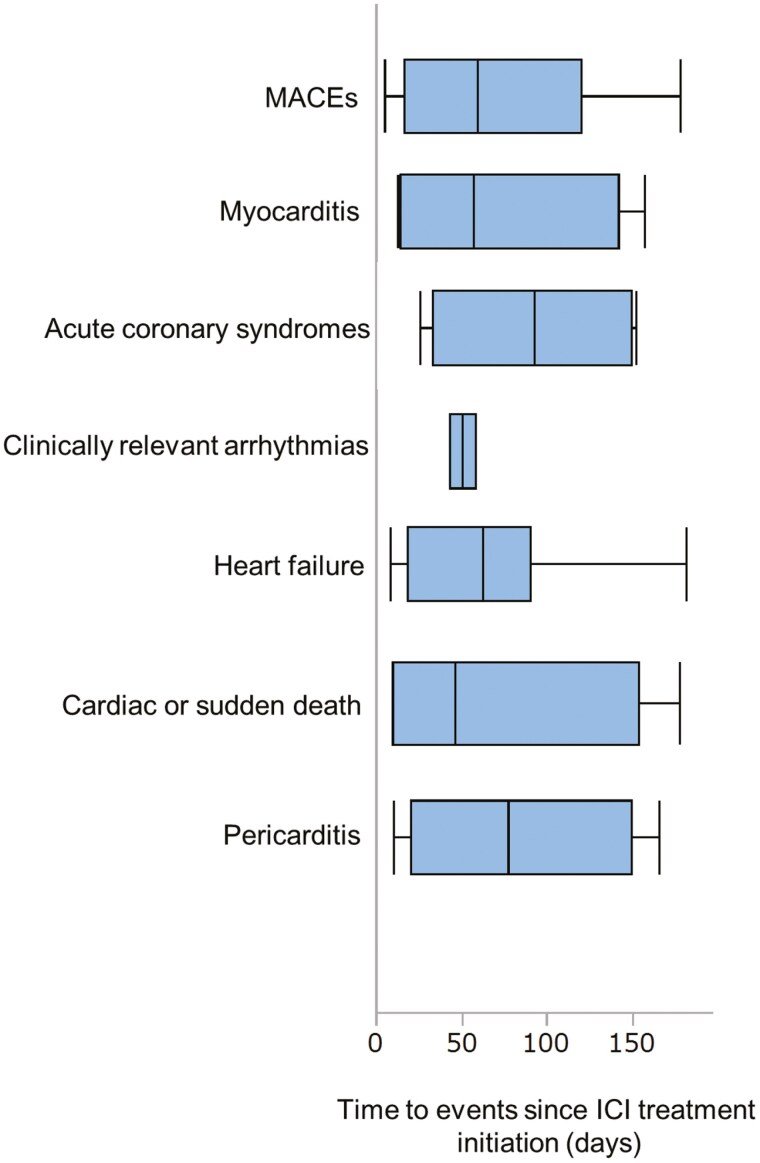
Time to events in the matched ICI cohort. Time to events is shown as the median number of days from the first ICI treatment initiation. The median time to onset for MACEs was 59 (IQR17-120) days, myocarditis 54 (IQR 12-138) days, ACS 94 (IQR 35-150) days, clinically relevant arrhythmias 55 (IQR 47-62) days, heart failure 59 (IQR 16-88) days, cardiac or sudden death 40 (IQR 7-136) days, and pericarditis 74 (IQR 18-146) days. Abbreviations: ACS, acute coronary syndromes; ICI, immune checkpoint inhibitor; IQR, interquartile range; MACEs, major adverse cardiovascular events.

**Figure 3.**
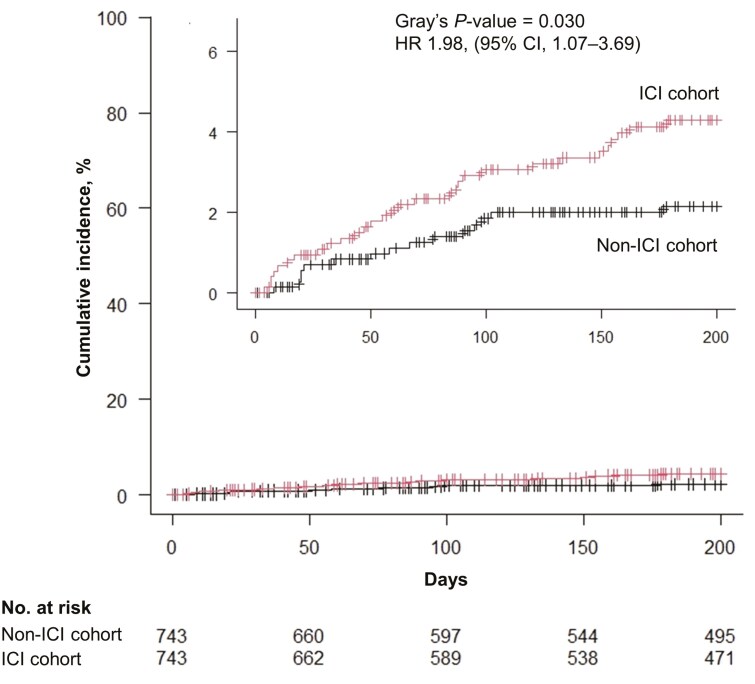
Cumulative incidence of MACEs in the matched ICI cohort versus the matched non-ICI cohort, considering non-cardiac and non-sudden deaths as competing risks. The incidence of MACEs in the matched ICI cohort was significantly higher than that observed in the matched non-ICI cohort. Abbreviations: CI, confidence interval; HR, hazard ratio; ICI, immune checkpoint inhibitor; MACEs, major adverse cardiovascular events

As shown in **Figure 2**, the time to events in the matched cohort is presented as the median number of days from the first ICI treatment initiation. The median time to MACE onset was 59 (IQR, 17-120) days. At 180 days, the matched cohort of patients receiving ICIs demonstrated a 4.0% incidence of MACEs, which was significantly higher than that observed in the matched non-ICI cohort (HR, 1.98; 95% CI, 1.07-3.69; *P*-value = 0.030) (Table 2**and Figure 3**). Among these events, heart failure occurred most frequently (1.7%), followed by acute coronary syndromes (0.8%), myocarditis (0.5%), cardiac or sudden death (0.5%), and pericarditis (0.5%). The median follow-up period was 329 (IQR, 147-625) days. A detailed analysis revealed that the cases of myocarditis and pericarditis were significantly increased (**Supplementary Figures S3****and****4**). However, as no patients in the non-ICI cohort developed myocarditis and pericarditis, the HRs for these conditions were not calculated. The incidence of acute coronary syndromes, clinically relevant arrhythmias, heart failure, or cardiac death/sudden death showed no significant difference (**Supplementary Figures S5****-****8**). A sensitivity analysis including smoking status and cancer stage in the propensity score model yielded an HR of 1.66 (95% CI: 0.92-3.00; *P*-value = 0.094) for MACEs, demonstrating a consistent trend toward higher incidence in the ICI cohort, consistent with the main analysis (**Supplementary Tables S6****and****7**). Moreover, multivariable Fine and Gray regression analysis also revealed that ICI showed a numerically higher but not statistically significant risk for developing MACEs at 180 days in the full study cohort (HR 1.68, 95% CI 0.99-2.86, *P*-value = .055) (**Supplementary Table S8**). A subgroup analysis comparing ICI plus platinum-based chemotherapy (carboplatin or cisplatin) to any non-ICI chemotherapy regimen did not show a statistically significant risk of MACEs (HR 1.19, 95% CI, 0.32-4.43; *P*-value = 0.79) (**Supplementary Tables S9****and****10**).

**Table 2.** HRs of MACEs and individual components in the matched ICI and non-ICI cohorts.

	Matched ICI cohort (*n* = 743)	Matched non-ICI cohort (*n* = 743)	HR (95% CI) ^b^^)^	*P*-value
MACEs ^a^^)^, *n* (%)	30 (4.0)	15 (2.0)	1.98 (1.07-3.69)	.030
Myocarditis, *n* (%)	4 (0.5)	0 (0.0)	N/A	.048
Acute coronary syndromes, *n* (%)	6 (0.8)	4 (0.5)	1.53 (0.44-5.30)	.50
Clinically relevant arrhythmias, *n* (%)	2 (0.3)	1 (0.1)	1.98 (0.18-21.84)	.58
Heart failure, *n* (%)	13 (1.7)	9 (1.2)	1.42 (0.61-3.33)	.42
Cardiac or sudden death, *n* (%)	4 (0.5)	2 (0.3)	1.48 (0.25-8.82)	.67
Pericarditis, *n* (%)	4 (0.5)	0 (0.0)	N/A	.048

Abbreviaitons: CI, confidence interval; HR, hazard ratio; ICI, immune checkpoint inhibitor; MACEs, major adverse cardiovascular events; N/A, not applicable.

^a)^MACEs include myocarditis, acute coronary syndromes, clinically relevant arrhythmias, heart failure, cardiac or sudden death, and pericarditis.

^b)^“Matched non-ICI cohort” was considered a reference category.

### Risk factors for cardiovascular events in the full ICI cohort

The results of the univariable and multivariable Fine and Gray regression analyses are presented in Tables 3 and 4**, and****Supplementary Table S11**. In the univariable analysis, although male sex, dyslipidemia, chronic renal failure, prior heart failure, and chronic liver disease appeared to increase the likelihood of MACE development, the multivariable analysis revealed that only chronic renal failure (HR, 2.16; 95% CI, 1.05-4.46; *P*-value = .038) and prior heart failure (HR, 3.08; 95% CI, 1.86-5.11; *P*-value < 0.001) remained significantly associated with MACE development.

**Table 3.** Univariable analysis of factors associated with MACEs and individual outcomes in the full ICI cohort.

Variables	MACEs ^a^^)^		Myocarditis		Acute coronary syndromes		Clinically relevant arrhythmias		Heart failure		Cardiac or sudden death		Pericarditis	
	HR (95% CI)	*P*-value	HR (95% CI)	*P*-value	HR (95% CI)	*P*-value	HR (95% CI)	*P*-value	HR (95% CI)	*P*-value	HR (95% CI)	*P*-value	HR (95% CI)	*P*-value
Age	1.00 (0.98-1.03)	.86	0.96 (0.90-1.01)	.13	1.00 (0.94-1.08)	.90	1.02 (0.93-1.13)	.64	1.02 (0.98-1.06)	.37	0.97 (0.91-1.04)	.44	0.96 (0.86-1.08)	.52
Sex (male)	1.77 (0.96-3.26)	.068	1.40 (0.15-13.4)	.77	0.93 (0.28-3.09)	.91	3.27 (0.40-26.54)	.27	1.67 (0.72-3.87)	.23	2.34 (0.27-19.97)	.44	0.39 (0.05-2.73)	.34
Body mass index	0.99 (0.93-1.06)	.86	1.02 (0.89-1.18)	.73	1.03 (0.89-1.19)	.71	0.94 (0.78-1.13)	.49	1.01 (0.94-1.10)	.73	0.79 (0.70-0.90)	<.001	0.91 (0.58-1.45)	.70
Diabetes	0.65 (0.28-1.51)	.31	2.03 (0.21-19.40)	.54	1.23 (0.27-5.60)	.79	N/A		0.63 (0.19-2.06)	.44	N/A		N/A	
Hypertension	1.31 (0.67-2.57)	.43	N/A		1.35 (0.30-6.10)	.70	0.96 (0.12-7.75)	.97	1.57 (0.65-3.80)	.32	N/A		2.35 (0.24-22.78)	.46
Dyslipidemia	2.35 (0.93-5.92)	.070	N/A		2.30 (0.29-17.99)	.43	8.88 (1.79-44.14)	.008	2.75 (0.83-9.11)	.10	N/A		N/A	
Chronic renal failure	2.44 (1.21-4.93)	.013	N/A		2.81 (0.62-12.80)	.18	N/A		2.02 (0.72-5.71)	.18	2.84 (0.33-24.1)	.34	4.65 (0.48-44.85)	.18
Prior myocardial infarction	N/A		N/A		N/A		N/A		N/A		N/A		N/A	
Prior heart failure	3.41 (2.07-5.63)	<.001	1.44 (0.15-13.70)	.75	1.44 (0.39-5.31)	.59	7.16 (1.71-29.91)	.007	6.43 (3.18-13.01)	<.001	N/A		1.40 (0.15-13.36)	.77
Chronic obstructive pulmonary disease	0.95 (0.41-2.21)	.91	2.97 (0.31 -28.63)	.35	N/A		5.73 (1.40-23.49)	.015	0.60 (0.14-2.51)	.48	N/A		N/A	
Chronic liver disease	1.47 (0.88-2.47)	.14	N/A		1.81 (0.58-5.68)	.31	2.48 (0.62-9.94)	.20	1.70 (0.84-3.44)	.14	0.49 (0.06-4.22)	.52	0.62 (0.07-5.54)	.67

Abbreviations: CI, confidence interval; HR, hazard ratio; ICI, immune checkpoint inhibitor; MACEs, major adverse cardiovascular events; N/A, not applicable.

^a)^MACEs include myocarditis, acute coronary syndromes, clinically relevant arrhythmias, heart failure, cardiac or sudden death, and pericarditis.

**Table 4.** Multivariable analysis of factors associated with MACEs in the full ICI cohort.

Variables	MACEs ^a^^)^	
	HR (95% CI)	*P*-value
Sex (male)	1.53 (0.83-2.83)	.17
Dyslipidemia	1.92 (0.75-4.91)	.17
Chronic renal failure	2.16 (1.05-4.46)	.038
Prior heart failure	3.08 (1.86-5.11)	<.001
Chronic liver disease	1.22 (0.72-2.06)	.46

Abbreviations: CI, confidence interval; HR, hazard ratio; ICI, immune checkpoint inhibitor; MACEs, major adverse cardiovascular events.

^a)^MACEs include myocarditis, acute coronary syndromes, clinically relevant arrhythmias, heart failure, cardiac or sudden death, and pericarditis.

## Discussion

To the best of our knowledge, this study represents one of the largest database analyses evaluating the impact of ICI treatment on cardiovascular adverse events in patients with primary lung cancer in an Asian population. The following were the main findings: (1) cardiovascular adverse events, although infrequent, were significantly increased in patients treated with ICIs compared with those receiving non-ICI chemotherapy; and (2) cardiovascular adverse events in patients treated with ICIs were associated with prior heart failure and concurrent chronic renal failure.

Our study validates existing evidence that ICI treatment is associated with the risk of MACEs compared with non-ICI chemotherapy in Asian populations, consistent with findings in Western populations.^11^ Although ICI use did not show a significant association with MACEs in the two sensitivity analyses, a trend toward a positive association was observed, suggesting a potential cardiovascular risk in patients with primary lung cancer. Among the individual components of MACEs, we noted evidence of an association between ICI use and myocarditis or pericarditis. The observed increase in myocarditis incidence is clinically noteworthy, as it is the most severe cardiovascular adverse event,^1^ typically occurring within 3-10 weeks of ICI treatment initiation,^16^ aligning with our study results (median, 54 [IQR, 12-138] days). The incidence of myocarditis in the matched ICI cohort was marginally lower (0.5%) than previously documented rates.^5,16^ This lower incidence may be due to the characteristics of this real-world database, which lacks routine prospective screening during and following ICI treatment, possibly omitting mild or asymptomatic cases. Another explanation could be that most of the participants in our study received ICI monotherapy; few were treated with dual ICIs, which pose a higher myocarditis risk than monotherapy.^17^ In contrast, our study showed no association between ICI plus platinum-based chemotherapy and MACE risk; however, previous studies have indicated a greater risk with combined ICI and chemotherapy regimens.^17^ This finding may be due to the small sample size of the subgroup receiving this specific regimen. In our study, among the MACE components, heart failure was the most common (1.7%), consistent with the results of previous studies.^5,18^ In patients receiving ICIs, nonmyocarditis-related heart failure with reduced ejection fraction emerged as the predominant late-onset cardiovascular adverse event, typically developing at 90 days or more following treatment initiation.^19^ Considering the established relationship between inflammation and heart failure development and progression, inhibition of immune checkpoints that mitigate proinflammatory responses could pose a risk of cardiac dysfunction, potentially leading to heart failure.^20^

Our study observed that cardiovascular adverse events in ICI-treated patients were associated with prior heart failure and chronic renal failure. Previous studies have identified the type of primary cancer; pre-existing cardiovascular conditions, including hypertension, diabetes mellitus, and acute coronary syndromes; female sex; treatment regimen; and advanced age as risk factors for ICI-related MACEs.^4,5,11,17^ However, in either Western or Asian populations treated with ICIs, chronic renal failure has not been previously identified as a risk factor for cardiovascular adverse events. The availability of blood test results in our database enabled the identification of chronic renal failure as a novel risk factor, which has significant clinical implications considering that patients with renal failure are frequently excluded from cancer clinical trials.^9^ Additionally, our study noted that the risk factors for ICI-related MACEs were consistent with those for cardiovascular diseases unrelated to ICIs. Various factors, including chronic renal failure and prior heart failure, were significant contributors to ICI-related MACEs and traditional cardiovascular diseases. This overlap suggests that patients with established cardiovascular risk profiles can be more susceptible to cardiovascular immune-related adverse events when undergoing ICI treatment. The overlapping risk factors underscore the significance of comprehensive cardiovascular risk assessment before ICI treatment initiation, as early identification and management of these factors may help mitigate the incidence and severity of ICI-related MACEs.

### Strengths and study limitations

This study had several limitations. First, the sample size was limited, and the observation period was brief; however, this study provided one of the largest sources of evidence available to date for Asian populations. Second, the outcomes assessed in this study were derived from routinely collected data based on ICD-10 code diagnoses and outcomes rather than actual diagnoses and outcomes determined by physicians. Therefore, some misclassifications of patients’ diseases and outcomes may be contained in the database. Third, the suitability of employing a 180-day fixed look-back period for estimating the comorbidity rates in the RWD database was not unequivocal. Fourth, due to the small number of events for each outcome, we could not conduct reliable regression analyses for each MACE component. Although multivariable regression identified prior heart failure and chronic renal failure as significant risk factors for overall MACEs, it remains unclear which specific cardiovascular outcomes were most influenced by these conditions. Fifth, this study used a nationwide Japanese hospital database without race or ethnicity information. Since most patients were likely of Japanese descent, direct comparisons with Western populations were not possible, and the findings should be interpreted accordingly. Finally, if key confounders, particularly unmeasured factors like performance status and frailty, were not accounted for in the propensity score matching, the comparability between the ICI and non-ICI cohorts may be compromised. Thus, the results should be interpreted with caution, given the study’s retrospective design and population heterogeneity. However, to address these limitations, we conducted sensitivity analyses and observed a similar trend in cardiovascular event incidence. Additionally, the inclusion of a large, diverse population of patients with primary lung cancer across multiple Japanese centers enhances the generalizability of our findings within the Asian context, representing a key strength of this study.

## Conclusions

Cardiovascular adverse events were infrequent but occurred significantly more often with ICI treatment compared to non-ICI chemotherapy. Although the overall incidence of MACEs was significantly higher in the ICI treatment, this was primarily due to inflammation-related events, such as myocarditis and pericarditis. In contrast, evidence regarding other MACEs components—including ACS, clinically relevant arrhythmias, heart failure, and cardiac or sudden death—remains inconclusive based on our current data. Furthermore, the observed increase in MACEs incidence among lung cancer patients with prior heart failure or chronic renal failure receiving ICI treatment underscores the need for larger clinical studies focusing on this high-risk subgroup to better assess the balance between the therapeutic benefits and cardiovascular risks of ICI treatment.

## Supplementary Material

oyaf151_suppl_Supplementary_Tables_1-11_Figures_1-8

## Data Availability

The data used to support the findings of this study are available from the corresponding author upon request.
