# Supplementary material for: Cardiovascular adverse events in patients with lung cancer treated with immune checkpoint inhibitors: a nationwide database study
Source: Oncologist. 2025 Jun 23;30(6):oyaf151. doi: 10.1093/oncolo/oyaf151 (PMC12205995; doi:10.1093/oncolo/oyaf151)
Supplement: oyaf151_suppl_Supplementary_Tables_1-11_Figures_1-8 [file oyaf151_suppl_supplementary_tables_1-11_figures_1-8.zip › Supplementary Tables.DOC]

**SUPPLEMENTARY MATERIAL**

**Table of Contents**

**1. Supplementary Table 1.**

**2. Supplementary Table 2.**

**3. Supplementary Table 3.**

**4. Supplementary Table 4.**

**5. Supplementary Table 5.**

**6. Supplementary Table 6.**

**7. Supplementary Table 7.**

**8. Supplementary Table 8.**

**9. Supplementary Table 9.**

**10. Supplementary Table 10.**

**11. Supplementary Table 11**

**Supplementary Table 1. The RECORD statement ^1^ – checklist of items, extended from the STROBE statement, that should be reported in observational studies using routinely collected health data.**

|  | **Item No.** | **STROBE statement items** | **Location in manuscript where items are reported** | **RECORD** **statement items** | **Location in manuscript where items are reported** |
| --- | --- | --- | --- | --- | --- |
| **Title and Abstract** | | | | | |
|  | 1 | (a) Indicate the study’s design with a commonly used term in the title or the abstract  (b) Provide in the abstract an informative and balanced summary of what was done and what was found | (a) Abstract  (b) Abstract | RECORD 1.1: The type of data used should be specified in the title or abstract. When possible, the name of the databases used should be included.  RECORD 1.2: If applicable, the geographic region and timeframe within which the study took place should be reported in the title or abstract.  RECORD 1.3: If linkage between databases was conducted for the study, this should be clearly stated in the title or abstract. | (1) Title, Abstract  (2) Abstract |
| **Introduction** | | | | | |
| Background rationale | 2 | Explain the scientific background and rationale for the investigation being reported | Introduction |  |  |
| Objectives | 3 | State specific objectives, including any prespecified hypotheses | Introduction |  |  |
| **Methods** | | | | | |
| Study Design | 4 | Present key elements of study design early in the paper | Materials and Methods |  |  |
| Setting | 5 | Describe the setting, locations, and relevant dates, including periods of recruitment, exposure, follow-up, and data collection | Materials and Methods |  |  |
| Participants | 6 | *(a) Cohort study* - Give the eligibility criteria, and the sources and methods of selection of participants. Describe methods of follow-up  *(b) Cohort study* - For matched studies, give matching criteria and number of exposed and unexposed | 1. Materials and Methods 2. Materials and Methods | RECORD 6.1: The methods of study population selection (such as codes or algorithms used to identify subjects) should be listed in detail. If this is not possible, an explanation should be provided.  RECORD 6.2: Any validation studies of the codes or algorithms used to select the population should be referenced. If validation was conducted for this study and not published elsewhere, detailed methods and results should be provided.  RECORD 6.3: If the study involved linkage of databases, consider use of a flow diagram or other graphical display to demonstrate the data linkage process, including the number of individuals with linked data at each stage. | (1) Materials and Methods  (2) Materials and Methods |
| Variables | 7 | Clearly define all outcomes, exposures, predictors, potential confounders, and effect modifiers. Give diagnostic criteria, if applicable. | Materials and Methods | RECORD 7.1: A complete list of codes and algorithms used to classify exposures, outcomes, confounders, and effect modifiers should be provided. If these cannot be reported, an explanation should be provided. | Supplementary Tables 2–3 |
| Data sources/ measurement | 8 | For each variable of interest, give sources of data and details of methods of assessment (measurement).  Describe comparability of assessment methods if there is more than one group | Materials and Methods |  |  |
| Bias | 9 | Describe any efforts to address potential sources of bias | Materials and Methods |  |  |
| Study size | 10 | Explain how the study size was arrived at | Figure 1, Supplementary Figure 1 |  |  |
| Quantitative variables | 11 | Explain how quantitative variables were handled in the analyses. If applicable, describe which groupings were chosen, and why | Materials and Methods |  |  |
| Statistical methods | 12 | (a) Describe all statistical methods, including those used to control for confounding  (b) Describe any methods used to examine subgroups and interactions  (c) Explain how missing data were addressed  (d) *Cohort study* - If applicable, explain how loss to follow-up was addressed  (e) Describe any sensitivity analyses | (a) Materials and Methods  (b) Materials and Methods  (c) Materials and Methods  (e) Materials and Methods |  |  |
| Data access and cleaning methods |  |  |  | RECORD 12.1: Authors should describe the extent to which the investigators had access to the database population used to create the study population.  RECORD 12.2: Authors should provide information on the data cleaning methods used in the study. | (1) Materials and Methods  (2) Identifying and correcting typographical and entry errors |
| Linkage |  |  |  | RECORD 12.3: State whether the study included person-level, institutional-level, or other data linkage across two or more databases. The methods of linkage and methods of linkage quality evaluation should be provided. | (3) No data linkage across databases was performed in this study. |
| **Results** | | | | | |
| Participants | 13 | (a) Report the numbers of individuals at each stage of the study (*e.g.*, numbers potentially eligible, examined for eligibility, confirmed eligible, included in the study, completing follow-up, and analysed)  (b) Give reasons for non-participation at each stage.  (c) Consider use of a flow diagram | (a) Materials and Methods, Figure 1  (b) Materials and Methods, Figure 1  (c) Materials and Methods, Figure 1 | RECORD 13.1: Describe in detail the selection of the persons included in the study (*i.e.,* study population selection) including filtering based on data quality, data availability and linkage. The selection of included persons can be described in the text and/or by means of the study flow diagram. | Materials and Methods, Figure 1 |
| Descriptive data | 14 | (a) Give characteristics of study participants (*e.g.*, demographic, clinical, social) and information on exposures and potential confounders  (b) Indicate the number of participants with missing data for each variable of interest  (c) *Cohort study* - summarise follow-up time (*e.g.*, average and total amount) | (a) Results, Table 1  (b) Results  (c) Results |  |  |
| Outcome data | 15 | *Cohort study* - Report numbers of outcome events or summary measures over time | Results, Table 2 |  |  |
| Main results | 16 | (a) Give unadjusted estimates and, if applicable, confounder-adjusted estimates and their precision (e.g., 95% confidence interval). Make clear which confounders were adjusted for and why they were included  (b) Report category boundaries when continuous variables were categorized  (c) If relevant, consider translating estimates of relative risk into absolute risk for a meaningful time period | (a) Results, Tables 3–4, Supplementary Table 5 |  |  |
| Other analyses | 17 | Report other analyses done—e.g., analyses of subgroups and interactions, and sensitivity analyses | Results, Supplementary Tables 6–11 |  |  |
| **Discussion** | | | | | |
| Key results | 18 | Summarise key results with reference to study objectives | Discussion |  |  |
| Limitations | 19 | Discuss limitations of the study, taking into account sources of potential bias or imprecision. Discuss both direction and magnitude of any potential bias | Discussion | RECORD 19.1: Discuss the implications of using data that were not created or collected to answer the specific research question(s). Include discussion of misclassification bias, unmeasured confounding, missing data, and changing eligibility over time, as they pertain to the study being reported. | Discussion |
| Interpretation | 20 | Give a cautious overall interpretation of results considering objectives, limitations, multiplicity of analyses, results from similar studies, and other relevant evidence | Discussion |  |  |
| Generalisability | 21 | Discuss the generalisability (external validity) of the study results | Discussion |  |  |
| **Other Information** | | | | | |
| Funding | 22 | Give the source of funding and the role of the funders for the present study and, if applicable, for the original study on which the present article is based | Funding section |  |  |
| Accessibility of protocol, raw data, and programming code |  |  |  | RECORD 22.1: Authors should provide information on how to access any supplemental information such as the study protocol, raw data, or programming code. | Data availability section |

[Reference] (1) Benchimol EI, Smeeth L, Guttmann A, et al. The REporting of studies Conducted using Observational Routinely-collected health Data (RECORD) Statement. PLoS Med. 2015;12(10):e1001885.

Checklist is protected under Creative Commons Attribution ([CC BY](http://creativecommons.org/licenses/by/4.0/)) license.

**Supplementary Table 2: Specification of ICI therapy and non-ICI chemotherapy by YJ codes, cancer type, and comorbidity definitions based on diagnoses by ICD-10 codes and/or JMP codes and/or YJ codes.**

| **ICIs** | **Definition based on YJ codes** |
| --- | --- |
| CTLA-4 inhibitor  Ipilimumab | 4291430A1026 (ipilimumab) |
| PD-1 inhibitor  Nivolumab  Pembrolizumab | 4291427A1024 (nivolumab), 4291427A2020 (nivolumab), 4291427A4023 (nivolumab), or 4291427A3027 (nivolumab)  4291435A2025 (pembrolizumab) |
| PD-L1 inhibitor  Atezolizumab  Durvalumab | 4291441A2020 (atezolizumab) or 4291441A1024 (atezolizumab)  4291443A1023 (durvalumab), 4291443A2020 (durvalumab) |
| ICI monotherapy | - The initial prescription of pembrolizumab, nivolumab, or atezolizumab in the database, without the administration of carboplatin or cisplatin on the index day or within 180 days following the index date. |
| ICI combined with chemotherapy | - The initial prescription of pembrolizumab, nivolumab, or atezolizumab in the database, administered concurrently with either carboplatin or cisplatin on the same day. |
| Dual ICI therapy | - The initial prescription of both nivolumab and ipilimumab in the database, administered concurrently with either carboplatin or cisplatin on the same day. |
| **Non-ICI anticancer drugs** | **Definition based on YJ codes** |
| Afatinib | 4291030F1020, 4291030F2027, 4291030F3023, or 4291030F4020 |
| Alectinib | 4291032M3021 |
| Bevacizumab | 4291413A1022 or 4291413A2029 |
| Carboplatin | 4291403A1088, 4291403A2084, 4291403A3080, 4291403A1061, 4291403A2068, 4291403A3064, 4291403A1053, 4291403A2050, 4291403A3056, 4291403A1118, 4291403A2114, 4291403A3110, 4291403A1096, 4291403A2092, 4291403A1126, 4291403A2122, 4291403A3129, or 4291403A3099 |
| Ceritinib | 4291044M1021 |
| Cisplatin | 4291401A1097, 4291401A2093, 4291401A3090, 4291401A1127, 4291401A2123, or 4291401A3120 |
| Crizotinib | 4291026M1023 or 4291026M2020 |
| Dabrafenib | 4291046M1020 or 4291046M2027 |
| Dacomitinib | 4291056F1025 or 4291056F2021 |
| Docetaxel | 4240405A3072 or 4240405A4079 |
| Entrectinib | 4291061M1025 or 4291061M2021 |
| Erlotinib | 4291016F1020 or 4291016F2027 |
| Etoposide | 4240001M2071 or 4240001M1075 |
| Gefitinib | 4291013F1027 |
| Gemcitabine | 4224403D1030 or 4224403D2037 |
| Irinotecan | 4240404A1040, 4240404A2047, 4240404A1091, or 4240404A2098 |
| Osimertinib | 4291045F1027 or 4291045F2023 |
| Paclitaxel | 4240406A1031, 4240406A2038, 4240406A1074, 4240406A2070, 4240406A1040, 4240406A2046, 4240406A1082, 4240406A2089, 4240406A1058, 4240406A2054, 4240406A3026, 4240406A1090, or 240406A2097 |
| Pemetrexed | 4229401D2026 or 4229401D1020 |
| Ramucirumab | 4291429A1023 or 4291429A2020 |
| Tegafur | 4229101F3037 or 4229101F4033 |
| Tepotinib | 4291065F1024 |
| Trametinib | 4291047F1026 or 4291047F2022 |
| Vinorelbine | 4240407A1028 or 4240407A2024 |
| Prior anthracycline use | - The presence of at least one claim for anthracycline-based chemotherapy within the 180-day look-back period before the cohort entry date. |
| Non-ICI chemotherapy | - The initial prescription of at least one non-ICI anti-cancer drug (as defined by any of the drugs listed above) recorded in the database, along with no documented ICI treatment during the 180-day look-back period or within 180 days following the index date. |
| **Cancer types** | **Definition based on ICD-10 codes** |
| Lung cancer | Satisfying both A) and B)   1. The presence of at least one of the following ICD-10 codes within 180 days prior to the index date:   C34.0, C34.1, C34.2, C34.3, or C34.9   1. The absence of the following ICD-10 code within 180 days prior to the index date:   C78.0 |
| A history of specific malignancies | - The presence of relevant ICD-10 codes, including those for multiple myeloma (C90), chronic myelogenous leukemia (C92), cervical cancer (C53), ovarian cancer (C56), hepatocellular carcinoma (C22), gastric cancer (C16), rectal cancer (C20), colon cancer (C18), malignant lymphoma (C83, C81, and C85), breast cancer (C50), and urothelial cancer (C67, C66, and C65), during the 180-day look-back period. |
| **Comorbidity** | **Definition based on ICD-10 codes and/or JMP codes and/or YJ codes** |
| Chronic liver disease | Satisfying A)   1. The presence of at least one of the following ICD-10 codes within 180 days prior to the index date:   ICD-10: B15, B16, B17, B18, B19, C22, K70, K71, K72, K73, K74, K75, K76, K77, Z94.4, or I98.2 |
| Chronic obstructive pulmonary disease | Satisfying both A) and B)   1. The presence of at least one of the following ICD-10 codes within 180 days prior to the index date:   ICD-10: J42, J43, or J44   1. The presence of at least one of the following YJ codes within 180 days prior to the index date: 2259709G1027, 2259807G1026, 2259807G2022, 2259805G1027, 2259806G1021, 2259806G2028, 2259710G1020, 2259805G1027, 2259808G1020, 2290805G1027, 2259712G1029, 2259808G1020, 2290805G1027, 2259713G1023, or 2259713G2020   Note: All the above-mentioned codes represent either long acting β2 agonists or long-acting anti-muscarinic agents. |
| Chronic renal failure | Satisfying both A) and B)   1. The presence of at least one of the following ICD-10 codes within 180 days prior to the index date:   ICD-10: E10.2, E11.2, E13.2, E14.2, I12.0, N02, N03, N04, N05, N06, N07, N08, N11, N12, N14, N18, N19, N26, N15.8, N15.9, N16.0, N16.2, N16.3, N16.4, N16.8, or Q61   1. Serum creatinine levels of 2.00 mg/dL or higher for men and 1.65 mg/dL or higher for women, recorded at least once within 180 days prior to the index date. |
| Diabetes mellitus | Satisfying both A) and B)   1. The presence of at least one of the following ICD-10 codes within 180 days prior to the index date:   ICD-10: E10, E11, E12, E13, or E14   1. The presence of at least one of the following YJ codes within 180 days prior to the index date: 3962002F2027, 3962002F3023, 3969007F1024, 3969007F2020, 3961003F1028, 3961003F2024, 3961008F6022, 3961008F4070, 3961008F5077, 3961007F1115, 3961007F2022, 3969006F1020, 3969006F2026, 3969008F1029, 3969008F2025, 3969013F1020, 3969013F2026, 3969010F4033, 3969010F1034, 3969010F2030, 3969010F3037, 3969011F1020, 3969012F3028, 3969012F2021, 3969012F1025, 3969014F1024, 3969015F1029, 3969015F2025, 3969016F1023, 3969017F1028, 3969017F2024, 3969024F2024, 3969025F1022, 3969025F2029, 3969003F3037, 3969003F4033, 3969004F1020, 3969004F2027, 3969009F6025, 3969009F4022, 3969009F5029, 3969018F1022, 3969018F2029, 3969019F1027, 3969019F2023, 3969020F1020, 3969020F2026, 3969021F1032, 3969022F1029, 3969023F1023, 3969023F2020, 3969107F1022, 3969100F1020, 3969100F2027, 3969101F1025, 3969101F2021, 3969106F1028, 3969103F1024, 3969103F2020, 3969105F1023, 3969104F1029, 3969104F2025, 3969102F1020, 2492415G6025, 2492415G1031, 2492415G3026, 2492414G9020, 2492414G5024, 2492414A1026, 2492413G8053, 2492403H5030, 2492413P1024, 2492419A1029, 2492416G2024, 2492421G1029, 2492418G1027, 3969108F1027, or 3969108F2023   Note: All the codes listed above indicate either oral hypoglycemic drugs or insulin. |
| Dyslipidemia | Satisfying both A) and B)   1. The presence of at least one of the following ICD-10 codes within 180 days prior to the index date:   ICD-10: E78   1. The presence of at least one of the following YJ codes within 180 days prior to the index date: 2189017F3025, 2189017F4021, 2189015F1120, 2189015F2127, 2189015F5029, 2189016F4027, 2189016F5023, 2189016F6020, 2189010F2027, 2189010F1039, 2189018F1027, 2189102F1024, 2189102F2020, 2189101F1020, or 2189101F2026   Note: The codes mentioned above represent lipid-lowering drugs, including statins and ezetimibe. |
| Hypertension | Satisfying both A) and B)   1. The presence of at least one of the following ICD-10 codes within 180 days prior to the index date:   ICD-10: I10, I11, I12, I13, or I15   1. The presence of at least one of the following YJ codes within 180 days prior to the index date: 2132003F3020, 2132003F1400, 2132003F3039, 2132003F1257, 2149012F1059, 2149012F2020, 2144001F1020, 2144001F2026, 2144001C1074, 2144012F1028, 2144012F2024, 2144008F1021, 2144008F2028, 2144002F1024, 2144002F2020, 2144002F3027, 2171022F6020, 2171022F4028, 2171022F3021, 2171014G3022, 2171014G4029, 2171014G5025, 2171014G3022, 2171014G4029, 2171014G5025, 2149048F3025, 2149048F1022, 2149048F2029, 2149044F7022, 2149044F6026, 2149044F5020, 2149042F1025, 2149042F2021, 2149042F3028, 2149040F1026, 2149040F2022, 2149040F3029, 2149040F4025, 2149046F1031, 2149046F2038, 2149046F3034, 2149041F5026, 2149041F6022, 2149041F7029, 2149041F8025, 2171006F1224, 2171006F2026, 2149026F1026, 2149026F2022, 2149026F3029, 2149026F4025, 2149037F1032, 2149037F2039, 2149037F3027, 2149043F1020, 2149043F2026, 2190101F1020, 2190102F1025, 2190103F1020, 2190104F1024, 2149117F1025, 2149117F2021, 2149114F1021, 2149115F1026, 2149115F2022, 2149116F1020, 2149116F2027, 2149118F1020, 2149118F2026, 2149120F1027, 2149110F1040, 2149110F2020, 2149121F1021, 2149121F2028, 2149112F1022, 2149112F2029, 2149111F1028, 2149111F2024, 2149119F1024, 2149119F2020, 2149113F1027, or 2149113F2023   Note: The YJ codes mentioned above represent antihypertensive drugs, including thiazides, angiotensin-converting enzyme inhibitors, angiotensin II receptor blockers, and calcium channel blockers. |
| Prior heart failure | Satisfying both A) and B)   1. The presence of at least one of the following ICD-10 codes within 180 days prior to the index date:   ICD-10: I50 or I11.0   1. The presence of at least one of the following JMP codes or one of the following YJ codes within 180 days prior to the index date:   ・JMP code: 160072510 (echocardiography)  ・YJ codes: 2119006F1020, 2119006F2026, 2119006F3022, 2139011F3026, 2139011F4022, 2139011D1022, 2190041F1027, 2190041F2023, 2190041F3020, 2139401A2137, 2139005F3039, 2139005F1052, 2139005F2342, 2133400D1082, 2133400D2097, 2133001F1522, 2133001F2057, 2133001C1097, 2149045F1029, 2149045F2025, 2149045F3021, 2190039F1020, 2190039F2026, 2190039F3022, 2119004F1055, 2119004F2051, 2119007D1031, 2119407A1020, or 2119407G1022  Note: The YJ codes mentioned above represent a variety of pharmacological classes, including loop diuretics (e.g., furosemide), mineralocorticoid receptor antagonists (e.g., spironolactone and eplerenone), vasopressin receptor antagonists (e.g., tolvaptan), inotropic agents (e.g., phosphodiesterase III inhibitors), heart rate modulators (e.g., ivabradine), and angiotensin receptor-neprilysin inhibitors (e.g., sacubitril/valsartan). |
| Prior myocardial infarction | Satisfying both A) and B)   1. The presence of the following ICD-10 code within 180 days prior to the index date:   ICD-10: I25.2   1. The presence of at least one of the following YJ codes within 180 days prior to the index date:   ・YJ codes: 3399007H1021, 3399103F1020, 3399102F1026, 1143010F2074, 3399008F2056, 3399008F3028, 3399101F1021, 3399009F1020, 3399009F3022, or 3399009F2026  Note: The YJ codes mentioned above represent antiplatelet drugs, including aspirin, clopidogrel, and prasugrel. |

Note: YJ codes serve as unique identifiers for each item included in the National Health Insurance (NHI) drug price standard, facilitating itemized claims in the Japanese healthcare insurance system. These codes allow for the precise identification of pharmaceutical items and can be queried on the following website: [https://www.iyaku.info/yjcode/](https://www.iyaku.info/yjcode/). In contrast, JMP codes categorize and standardize medical procedures, treatments, and services for billing and reimbursement purposes under the NHI. Each procedure or service is assigned a unique code that specifies the reimbursable amount, thus ensuring consistent pricing and streamlined tracking for healthcare providers and insurers throughout Japan. JMP codes can be accessed via the following website: [https://shinryohoshu.mhlw.go.jp/shinryohoshu/searchMenu/doSearchInputSp](https://shinryohoshu.mhlw.go.jp/shinryohoshu/searchMenu/doSearchInputSp).

For the ICI cohort, the index date was defined as the date of the first ICI administration recorded in the database. For the non-ICI cohort, the index date was defined as the date of the first prescription of the specific non-ICI anticancer drugs recorded in the database.

CTLA-4; cytotoxic T-lymphocyte-associated protein 4, ICD; international classification of diseases, ICI; immune checkpoint inhibitor, JMP; Japan-specific medical procedure, PD-1; programmed cell death 1, PD-L1; programmed death-ligand 1

**Supplementary Table 3:**  **Specification of outcomes definitions based on diagnoses by ICD-10 codes and/or JMP codes and/or YJ codes.**

| **Outcomes** | **Definition based on ICD-10 codes and JMA codes and/or YJ codes** |
| --- | --- |
| Acute coronary syndromes | Satisfying both A) and B)   1. The presence of at least one of the following ICD-10 codes after the index date:   ICD-10: I21.0, I21.1, I21.2, I21.3, I21.4, I21.9, I23.0, I23.2, I23.3, I23.4, I23.5, I20.1, I20.0, I20.9, or I22   1. The presence of at least one of the following JMP codes or at least one of the following YJ codes within 30 days after the ICD-10 code entry:   ・JMP codes:150374910 (plain old balloon angioplasty alone in percutaneous coronary intervention for acute myocardial infarction), 150375010 (plain old balloon angioplasty alone in percutaneous coronary intervention for unstable angina pectoris), 150375210 (stent use in percutaneous coronary intervention for acute myocardial infarction), 150375310 (stent use in percutaneous coronary intervention for unstable angina pecoris), 150375410 (percutaneous coronary intervention using devices other than stents or balloons), 150375110 (thrombectomy), 150318310 (coronary artery bypass grafting for single vessel disease), 150145910 (coronary artery bypass grafting for multivessel disease), 150146010 (spasm provocation test)  ・YJ codes: 2171006F1224 (diltiazem), 2171006F2026 (diltiazem), 2171022F6020 (amlodipine), 2171022F4028 (amlodipine), 2171022F3021 (amlodipine), 2171014G3022 (nifedipine), 2171014G4029 (nifedipine), 2171014G5025 (nifedipine), 2171014G3022 (benidipine), 2171014G4029 (benidipine), or 2171014G5025 (benidipine) |
| Clinically relevant arrhythmias | Satisfying both A) and B)   1. The presence of at least one of the following ICD-10 codes after the index date:   ICD-10: I49.0, I48.0, I47.1, I44.1, I44.2, or I47.2   1. The presence of at least one of the following JMP codes or at least one of the following YJ codes within 30 days after the ICD-10 code entry:   ・JMP codes: 150267310 (tempprary pacemaker), 150140210 (PMI), 150395350 (leadless pacemaker), 150415210 (cardiac resynchronization therapy), 150387410 (transvenous implantable cardioverter-defibrillator), 150383250 (subcutaneous implantable cardioverter-defibrillator), 150415910 (cardiac resynchronization therapy), 140010310 (cardioversion and defibrillation), 140051410 (automated external defibrillator), 150262810 (catheter ablation without trans-septal catheterization), or 150346710 (trans-septal catheterization for catheter ablation)  ・YJ codes: 3992400A1123 (adenosine triphosphate), 3992400A2138 (adenosine triphosphate), 3992400A3045 (adenosine triphosphate), 2129410A1028 (amiodarone), 2129407D1030 (nifekalant), 2129013F2022 (sotalol), 2129013F1026 (sotalol), 2129011F1035 (bepridil), 2129011F2031 (bepridil), 2129402A1040 (verapamil), 2123404D1033 (landiolol), 2123404D3028 (landiolol), 2123011F2437 (atenolol), 2123011F1155 (atenolol), 2123016F3037 (bisoprolol), 2123016F1107 (bisoprolol), 2123016F2189 (bisoprolol), 2149010F1025 (metoprolol), 2149010F1173 (metoprolol), 2149010F1033 (metoprolol), 2149010F2072 (metoprolol), 2123011F1295 (atenolol), 2123011F2550 (atenolol), 1244400A1030 (magnesium sulfate), 2113003F2061 (digoxin), 2113003F1090 (digoxin), 2113400A1032 (digoxin), 2113005F1030 (metildigoxin), or 2113005F2028 (metildigoxin) |
| Cardiac or sudden death | Satisfying both A) and B)   1. The presence of at least one of the following ICD-10 codes after the index date:   ICD-10:R96.0, R99, I46.9, I46.0, or I46.1   1. The presence of at least one of the following JMP codes within 7 days after the ICD-10 code entry: 140010210 (cardiac massage), 150148010 (intra-aortic balloon pumping), 150395450 (Impella device), 150262910 (extracorporeal membrane oxygenation), or 150147770 (cardio-pulmonary bypass) |
| Heart failure | Satisfying both A) and B)   1. The presence of at least one of the following ICD-10 codes after the index date:   ICD-10: J81, I11.0, I50.1, I50.0, I50.9, R57.0, or I42.0   1. The presence of at least one of the following JMP codes or at least one of the following YJ codes within 30 days after the ICD-10 code entry:   ・JMP codes: 114006810 (mechanical ventilator), 40009310 (mechanical ventilator), 140023510 (mechanical ventilator), 114009610 (mechanical ventilator), 140039550 (mechanical ventilator), 140039650(mechanical ventilator), 150148010 (IABP), 150395450 (Impella device), 150262910 (extracorporeal membrane oxygenation), 114041210 (noninvasive positive pressure ventilation), or 114041310 (noninvasive positive pressure ventilation)  ・YJ codes: 2451400A1030 (adrenaline), 2119404G3056 (dobutamine), 2119404G4052 (dobutamine), 2119402P3030 (dopamine), 2119402P4037 (dopamine), 2119402P5025 (dopamine), 2451401A1034(noradrenaline), 2119408A1024 (milrinone), 2119006F1020 (pimobendan), 2119006F2026 (pimobendan), 2119006F3022(pimobendan), 2123016F3037 (bisoprolol), 2123016F1107(bisoprolol), 2123016F2189(bisoprolol), 2149032F3024 (carvedilol), 2149032F4020, (carvedilol), 2149032F1021(carvedilol), 2139011F3026 (tolvaptan), 2139011F4022 (tolvaptan), 2139011D1022 (tolvaptan), 2190041F1027 (sacubitril valsartan), 2190041F2023 (sacubitril valsartan), 2190041F3020 (sacubitril valsartan), 2139401A2137 (furosemide), 2139005F3039 (furosemide), 2139005F1052 (furosemide), 2139005F2342 (furosemide), 133400D1082 (potassium canrenoate), 2133400D2097 (potassium canrenoate), 2133001F1522 (spironolactone) 2133001F2057 (spironolactone), 2133001C1097(spironolactone), 2149045F1029 (eplerenone), 2149045F2025 (eplerenone), 2149045F3021 (eplerenone), 2190039F1020 (ivabradine), 2190039F2026 (ivabradine), 2190039F3022 (ivabradine), 2179400D1022 (carperitide), 2171403A7046 (nitroglycerin), 2171403A4055 (nitroglycerin), 2119004F1055 (Denopamine), 2119004F2051(denopamine), 2119007D1031 (docarpamine), 2119407A1020 (olprinone), or 2119407G1022 (olprinone) |
| Myocarditis | Satisfying both A) and B)   1. The presence of at least one of the following ICD-10 codes after the index date:   ICD-10: I40.8, I40.9, or I51.4   1. The presence of at least one of the following JMP codes or the elevated troponin level within 30 days after the ICD-10 code entry:   ・160188310 (myocardial biopsy)  ・170027870 (cardiac magnetic resonance imaging)  ・160072510 (echocardiography)  ・Troponin T > 0.1 ng/mL (100 pg/mL), Troponin I > 0.04 ng/mL (40 pg/mL), or a positive result on a qualitative test for troponin T or I. |
| Pericarditis | Satisfying both A) and B)   1. The presence of at least one of the following ICD-10 codes after the index date:   ICD-10: I30.9, I30.0, or I31.9   1. The presence of at least one of the following JMP codes or at least one of the following YJ codes within 30 days after the ICD-10 code entry:   ・JMP codes: 140010510 (pericardiocentesis)  ・YJ codes: 1149001F1463 (ibuprofen), 1149001F2206 (ibuprofen), 2649735Q1020 (loxoprofen), 1149019F1560 (loxoprofen), 1149019C1149 (loxoprofen), 1147002F1560 (diclofenac), 1149037F1186 (celecoxib), 1149037F2182 (celecoxib), 1149032F1060 (etodolac), 1149032F2147 (etodolac), 1149035F1136 (meloxicam), 1149035F2132 (meloxicam), 1141001X1045 (acetaminophen), 1141007F1209 (acetaminophen), 1141007R2031 (acetaminophen), 1141007F1195 (acetaminophen), 1141007F3031 (acetaminophen), 1141007F2051 (acetaminophen), 1141007F1195 (acetaminophen), 1141007F2043 (acetaminophen), 1141007F1152 (acetaminophen), or 1141007F1217 (acetaminophen) |

Note: YJ codes serve as unique identifiers for each item included in the National Health Insurance (NHI) drug price standard, facilitating itemized claims in the Japanese healthcare insurance system. These codes allow for the precise identification of pharmaceutical items and can be queried on the following website: <https://www.iyaku.info/yjcode/>. In contrast, JMP codes categorize and standardize medical procedures, treatments, and services for billing and reimbursement purposes under the NHI. Each procedure or service is assigned a unique code that specifies the reimbursable amount, thus ensuring consistent pricing and streamlined tracking for healthcare providers and insurers throughout Japan. JMP codes can be accessed via the following website: <https://shinryohoshu.mhlw.go.jp/shinryohoshu/searchMenu/doSearchInputSp>.

For the ICI cohort, the index date was defined as the date of the first ICI administration recorded in the database. For the non-ICI cohort, the index date was defined as the date of the first prescription of the specific non-ICI anticancer drugs recorded in the database.

ICD; international classification of diseases, ICI; immune checkpoint inhibitor, JMP; Japan-specific medical procedure

**Supplementary Table 4. ICI treatment in the full ICI cohort and the matched ICI cohort.**

| **Variables** | **Full ICI cohort**  **(n=936)** | **Matched ICI cohort**  **(n=743)** | **SMD ^a)^** |
| --- | --- | --- | --- |
| ICI and carboplatin or cisplatin, *n* (%) | 198 (21.2) | 150 (20.2) | 0.02 |
| Dual ICI therapy, *n* (%) | 3 (0.3) | 2 (0.3) | 0.01 |
| Nivolumab, *n* (%) | 306 (32.7) | 245 (33.0) | 0.01 |
| Pembrolizumab, *n* (%) | 412 (44.0) | 335 (45.1) | 0.02 |
| Atezolizumab, *n* (%) | 161 (17.2) | 122 (16.4) | 0.02 |
| Durvalumab, *n* (%) | 57 (6.1) | 41 (5.5) | 0.02 |
| Ipilimumab, *n* (%) | 3 (0.3) | 2 (0.3) | 0.01 |
| Firstline of ICI treatment, *n* (%) | 738 (78.8) | 574 (77.3) | 0.04 |

ICI, immune checkpoint inhibitor; SMD, standardized mean difference

^a)^An absolute SMD of ≤0.10 indicates a negligible difference in the measured variables between the cohorts.

**Supplementary Table 5. HRs of MACEs and individual components in the full ICI and non-ICI cohorts.**

|  | **Full ICI cohort**  **(n=936)** | **Full non-ICI cohort** ^b)^  **(n=1121)** | **HR (95% CI)** | ***P*-value** |
| --- | --- | --- | --- | --- |
| MACEs ^a)^, *n* (%) | 35 (3.7) | 27 (2.4) | 1.53 (0.92–2.52) | 0.099 |
| Myocarditis, *n* (%) | 4 (0.4) | 0 (0.0) | N/A | 0.030 |
| Acute coronary syndromes, *n* (%) | 6 (0.6) | 6 (0.5) | 1.19 (0.39–3.66) | 0.76 |
| Clinically relevant arrhythmias, *n* (%) | 3 (0.3) | 5 (0.4) | 0.70 (0.17–2.94) | 0.63 |
| Heart failure, *n* (%) | 15 (1.6) | 17 (1.5) | 1.04 (0.52–2.07) | 0.92 |
| Cardiac or sudden death, *n* (%) | 6 (0.6) | 3 (0.3) | 2.34 (0.43–12.72) | 0.33 |
| Pericarditis, *n* (%) | 4 ( 0.4) | 1 ( 0.1) | 4.69 (0.52–41.85) | 0.17 |

CI, confidence interval; HR, hazard ratio; ICI, immune checkpoint inhibitor; MACEs, major adverse cardiovascular events; N/A, not applicable

^a)^ MACEs include myocarditis, acute coronary syndromes, clinically relevant arrhythmias, heart failure, cardiac or sudden death, and pericarditis.

^b)^ ‘Full non-ICI cohort’ was considered a reference category.

**Supplementary Table 6. Baseline characteristics of patients before and after propensity-score matching, including adjustment for smoking status and cancer stage in addition to covariates used in the main analysis.**

| **Variables** | **Before propensity-score matching** | | | **After propensity-score matching ^a)^** | | |
| --- | --- | --- | --- | --- | --- | --- |
|  | **Full ICI cohort**  **(n=936)** | **Full non-ICI cohort**  **(n=1121)** | **SMD ^b)^** | **Matched ICI cohort**  **(n=768)** | **Matched non-ICI cohort**  **(n=768)** | **SMD ^b)^** |
| Age, median (IQR), years | 71.0 (66.0–76.0) | 73.0 (68.0–79.0) | 0.23 | 71.0 (66.0–77.0) | 72.0 (66.0–77.0) | 0.02 |
| Sex (male), *n* (%) | 692 (73.9) | 232 (62.8) | 0.24 | 566 (73.7) | 576 (75.0) | 0.03 |
| Body mass index, median (IQR), kg/m^2^ | 21.8 (19.4–24.2) | 22.1 (20.0–24.3) | 0.09 | 22.1 (19.9–24.1) | 22.1 (20.3–23.5) | 0.02 |
| Diabetes, *n* (%) | 146 (15.6) | 140 (12.5) | 0.09 | 124 (16.1) | 101 (13.2) | 0.09 |
| Hypertension, *n* (%) | 103 (11.0) | 153 (13.7) | 0.08 | 85 (11.1) | 98 (12.8) | 0.05 |
| Dyslipidemia, *n* (%) | 25 (2.7) | 50 (4.5) | 0.10 | 24 (3.1) | 34 (4.4) | 0.07 |
| Chronic renal failure, *n* (%) | 84 (9.0) | 52 (4.6) | 0.17 | 51 (6.6) | 47 (6.1) | 0.02 |
| Prior myocardial infarction, *n* (%) | 22 (2.4) | 33 (2.9) | 0.04 | 19 (2.5) | 20 (2.6) | 0.01 |
| Prior heart failure, *n* (%) | 164 (17.5) | 217 (19.4) | 0.05 | 147 (19.1) | 138 (18.0) | 0.03 |
| Chronic obstructive pulmonary disease, *n* (%) | 110 (11.8) | 94 (8.4) | 0.11 | 71 (9.2) | 72 (9.4) | <0.01 |
| Chronic liver disease, *n* (%) | 271 (29.0) | 318 (28.4) | 0.01 | 225 (29.3) | 225 (29.3) | <0.01 |
| Current or former smoker, *n* (%) | 722 (77.1) | 768 (68.5) | 0.20 | 583 (75.9) | 584 (76.0) | <0.01 |
| Cancer stage IV, *n* (%) | 417 (53.1) | 519 (40.8) | 0.24 | 302 (39.3) | 302 (39.3) | <0.01 |

ICI, immune checkpoint inhibitor; IQR, interquartile range; SMD, standardized mean difference

^a)^ Patients were matched on the basis of a 1:1 ratio, using a propensity score-based procedure involving age, sex, body mass index, prior myocardial infarction, prior heart failure, chronic renal failure, smoking status, and cancer stage. The caliper used for matching was set at 0.2.

^b)^ An absolute SMD of ≤0.10 indicates a negligible difference in the measured variables between the cohorts.

**Supplementary Table 7. HRs of MACEs and individual components in the matched ICI and non-ICI cohorts.**

|  | **Matched ICI cohort**  **(n=768)** | **Matched non-ICI cohort**  **(n=768)** | **HR (95% CI)** ^b)^ | ***P*-value** |
| --- | --- | --- | --- | --- |
| MACEs ^a)^, *n* (%) | 28 (3.6) | 16 (2.1) | 1.66 (0.92–3.00) | 0.094 |
| Myocarditis, *n* (%) | 3 (0.4) | 0 (0) | N/A | 0.087 |
| Acute coronary syndromes, *n* (%) | 6 (0.8) | 2 (0.3) | 2.92 (0.59–14.47) | 0.19 |
| Clinically relevant arrhythmias, *n* (%) | 1 (0.1) | 4 (0.5) | 0.24 (0.03–2.17) | 0.20 |
| Heart failure, *n* (%) | 15 (2.0) | 11 (1.4) | 1.34 (0.61–2.90) | 0.47 |
| Cardiac or sudden death, *n* (%) | 3 (0.4) | 2 (0.3) | 1.96 (0.18–21.6) | 0.58 |
| Pericarditis, *n* (%) | 3 (0.4) | 0 (0) | N/A | 0.087 |

CI, confidence interval; HR, hazard ratio; ICI, immune checkpoint inhibitor; MACEs, major adverse cardiovascular events; N/A, not applicable

^a)^ MACEs include myocarditis, acute coronary syndromes, clinically relevant arrhythmias, heart failure, cardiac or sudden death, and pericarditis.

^b)^ ‘Matched non-ICI cohort’ was considered a reference category.

**Supplementary Table 8.** **Multivariable analysis of factors associated with MACEs in the full study cohort.**

| **Variables** | **MACEs ^a)^** | |
| --- | --- | --- |
|  | **HR (95% CI)** | ***P*-value** |
| ICI | 1.68 (0.99–2.86) | 0.055 |
| Age | 1.00 (0.97–1.03) | 0.80 |
| Sex (male) | 1.63 (0.85–3.15) | 0.14 |
| Body mass index | 0.99 (0.92–1.06) | 0.71 |
| Chronic renal failure | 2.30 (1.13– 4.71) | 0.022 |
| Prior myocardial infarction | N/A | N/A |
| Prior heart failure | 2.96 (1.74–5.03) | < 0.001 |

CI, confidence interval; HR, hazard ratio; ICI, immune checkpoint inhibitor; MACEs, major adverse cardiovascular events; N/A, not applicable

^a)^ MACEs include myocarditis, acute coronary syndromes, clinically relevant arrhythmias, heart failure, cardiac or sudden death, and pericarditis.

**Supplementary Table 9. Baseline patient characteristics before and after propensity-score matching.**

| **Variables** | **Before propensity-score matching** | | | **After propensity-score matching ^a)^** | | |
| --- | --- | --- | --- | --- | --- | --- |
|  | **ICI plus platinum-based chemotherapy cohort**  **(n=198)** | **non-ICI cohort**  **(n=1121)** | **SMD ^b)^** | **Matched ICI plus platinum-based chemotherapy cohort**  **(n=173)** | **Matched non-ICI cohort**  **(n=173)** | **SMD ^b)^** |
| Age, median (IQR), years | 71.0 (65.0–75.0) | 73.0 (68.0–79.0) | 0.30 | 70.0 (65.0–75.0) | 71.0 (65.0–77.0) | 0.01 |
| Sex (male), *n* (%) | 146 (73.7) | 232 (62.8) | 0.24 | 126 (72.8) | 133 (76.9) | 0.09 |
| Body mass index, median (IQR), kg/m^2^ | 22.6 (20.3–24.5) | 22.1 (20.0–24.3) | 0.11 | 22.6 (20.3–24.5) | 22.4 (20.7–24.3) | 0.05 |
| Diabetes, *n* (%) | 39 (19.7) | 140 (12.5) | 0.20 | 35 (20.2) | 33 (19.1) | 0.03 |
| Hypertension, *n* (%) | 20 (10.1) | 153 (13.6) | 0.11 | 16 (9.2) | 25 (14.5) | 0.16 |
| Dyslipidemia, *n* (%) | 5 (2.5) | 50 (4.5) | 0.10 | 5 (2.9) | 8 (4.6) | 0.09 |
| Chronic renal failure, *n* (%) | 19 (9.6) | 52 (4.6) | 0.19 | 14 (8.1) | 14 (8.1) | < 0.01 |
| Prior myocardial infarction, *n* (%) | 3 (1.5) | 33 (2.9) | 0.01 | 2 (1.2) | 1 (0.6) | 0.06 |
| Prior heart failure, *n* (%) | 42 (21.2) | 217 (19.4) | 0.05 | 39 (22.5) | 41 (23.7) | 0.03 |
| Chronic obstructive pulmonary disease, *n* (%) | 19 (9.6) | 94 (8.4) | 0.04 | 17 (9.8) | 22 (12.7) | 0.09 |
| Chronic liver disease, *n* (%) | 85 (42.9) | 318 (28.4) | 0.31 | 222 (29.9) | 209 (28.1) | 0.04 |

ICI, immune checkpoint inhibitor; IQR, interquartile range; SMD, standardized mean difference

^a)^ Patients were matched on the basis of a 1:1 ratio, using a propensity score-based procedure involving age, sex, body mass index, prior myocardial infarction, prior heart failure, and chronic renal failure. The caliper used for matching was set at 0.2.

^b)^ An absolute SMD of ≤0.10 indicates a negligible difference in the measured variables between the cohorts.

**Supplementary Table 10. HRs of MACEs and their individual components in the matched ICI plus platinum-based chemotherapy cohort and the matched non-ICI cohort.**

|  | **Matched ICI plus platinum-based chemotherapy cohort**  **(n=173)** | **Matched non-ICI cohort** ^b)^  **(n=173)** | **HR (95% CI)** | ***P*-value** |
| --- | --- | --- | --- | --- |
| MACEs ^a)^, *n* (%) | 5 (2.9) | 4 (2.3) | 1.19 (0.32–4.43) | 0.79 |
| Myocarditis, *n* (%) | 0 (0) | 0 (0) | N/A | N/A |
| Acute coronary syndromes, *n* (%) | 2 (1.2) | 1 (0.6) | 3.10 (0.29–33.07) | 0.35 |
| Clinically relevant arrhythmias, *n* (%) | 0 (0) | 0 (0) | N/A | N/A |
| Heart failure, *n* (%) | 1 (0.6) | 3 (1.7) | 0.32 (0.03–3.04) | 0.32 |
| Cardiac or sudden death, *n* (%) | 2 (1.2) | 0 (0) | N/A | 0.33 |
| Pericarditis, *n* (%) | 0 (0) | 0 (0) | N/A | N/A |

CI, confidence interval; HR, hazard ratio; ICI, immune checkpoint inhibitor; MACEs, major adverse cardiovascular events; N/A, not applicable

^a)^ MACEs include myocarditis, acute coronary syndromes, clinically relevant arrhythmias, heart failure, cardiac or sudden death, and pericarditis.

^b)^ ‘Matched non-ICI cohort’ was considered a reference category.

**Supplementary Table 11. Multivariable analysis of factors associated with clinically relevant arrhythmias and heart failure in the full ICI cohort.**

| **Variables** | **Clinically relevant arrhythmias** | |
| --- | --- | --- |
|  | **HR (95% CI)** | ***P*-value** |
| Dyslipidemia | 7.12 (1.31–38.70) | 0.023 |
| Prior heart failure | 4.75 (1.11–20.32) | 0.036 |
| Chronic obstructive pulmonary disease | 3.56 (0.94–13.45) | 0.062 |
| Chronic liver disease | 1.97 (0.44–8.82) | 0.38 |

CI, confidence interval; HR, hazard ratio; ICI, immune checkpoint inhibitor

| **Variables** | **Heart failure** | |
| --- | --- | --- |
|  | **HR (95% CI)** | ***P*-value** |
| Sex (male) | 1.33 (0.59–3.00) | 0.50 |
| Dyslipidemia | 2.10 (0.63–7.05) | 0.23 |
| Chronic renal failure | 1.74 (0.61–4.99) | 0.30 |
| Prior heart failure | 5.84 (2.94–11.60) | < 0.001 |
| Chronic liver disease | 1.32 (0.64–2.71) | 0.46 |

CI, confidence interval; HR, hazard ratio; ICI, immune checkpoint inhibitor
