# Supplementary figures and images for: Cardiovascular adverse events in patients with lung cancer treated with immune checkpoint inhibitors: a nationwide database study
Source: Oncologist. 2025 Jun 23;30(6):oyaf151. doi: 10.1093/oncolo/oyaf151 (PMC12205995; doi:10.1093/oncolo/oyaf151)

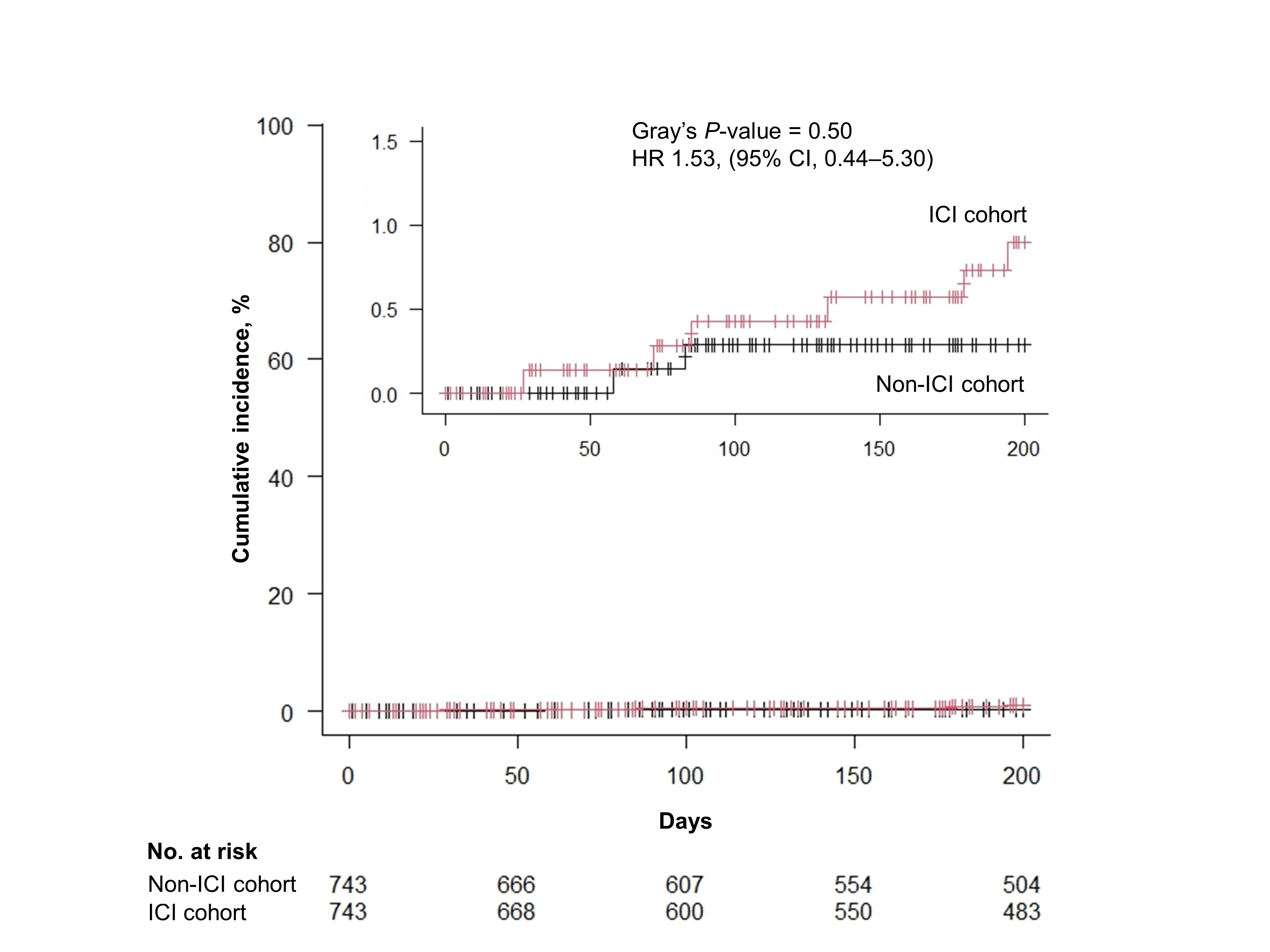

Supplement: oyaf151_suppl_Supplementary_Tables_1-11_Figures_1-8 [file oyaf151_suppl_supplementary_tables_1-11_figures_1-8.zip › Supplementary Figure 5.tif]

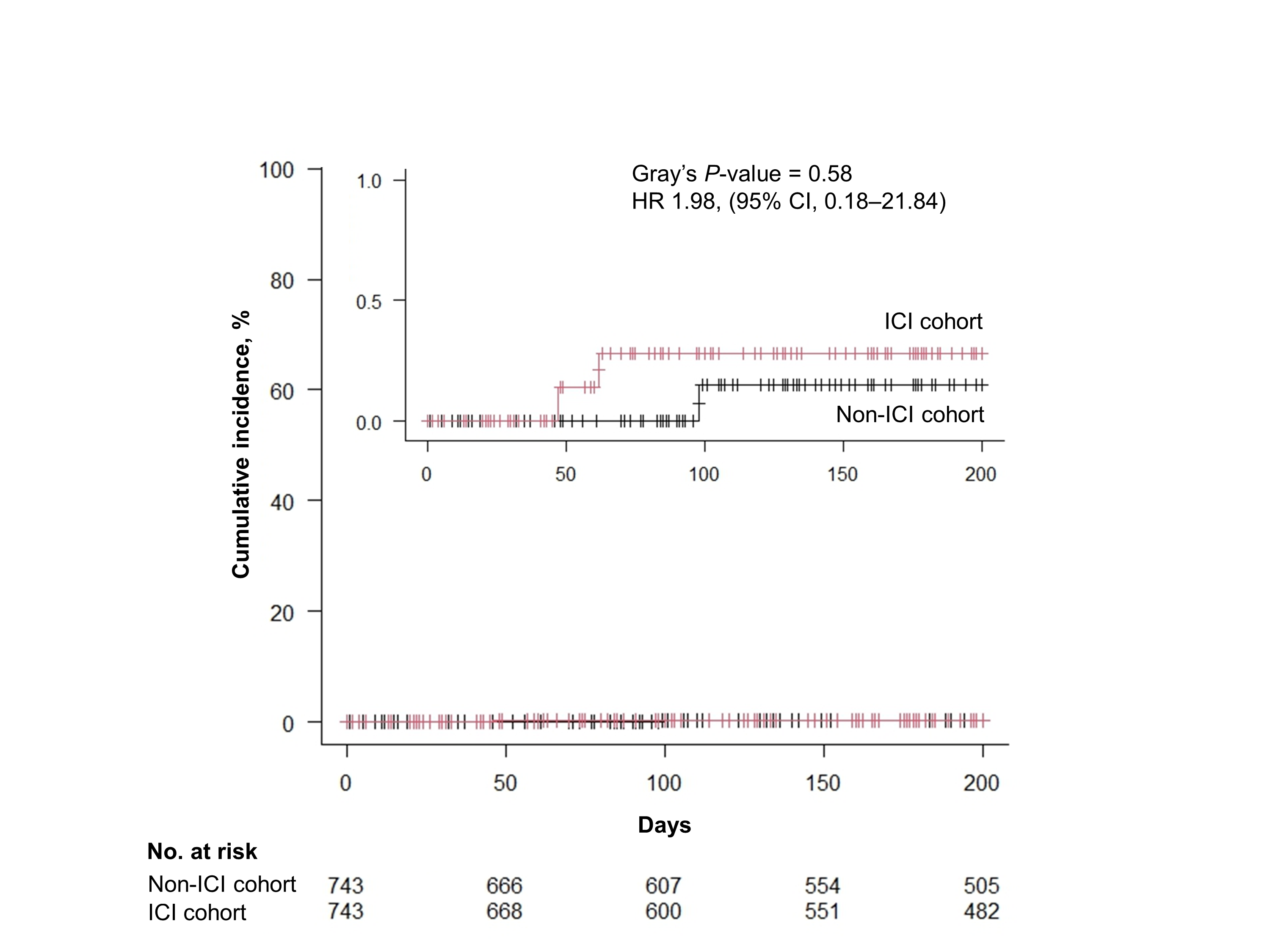

Supplement: oyaf151_suppl_Supplementary_Tables_1-11_Figures_1-8 [file oyaf151_suppl_supplementary_tables_1-11_figures_1-8.zip › Supplementary Figure 6.tif]

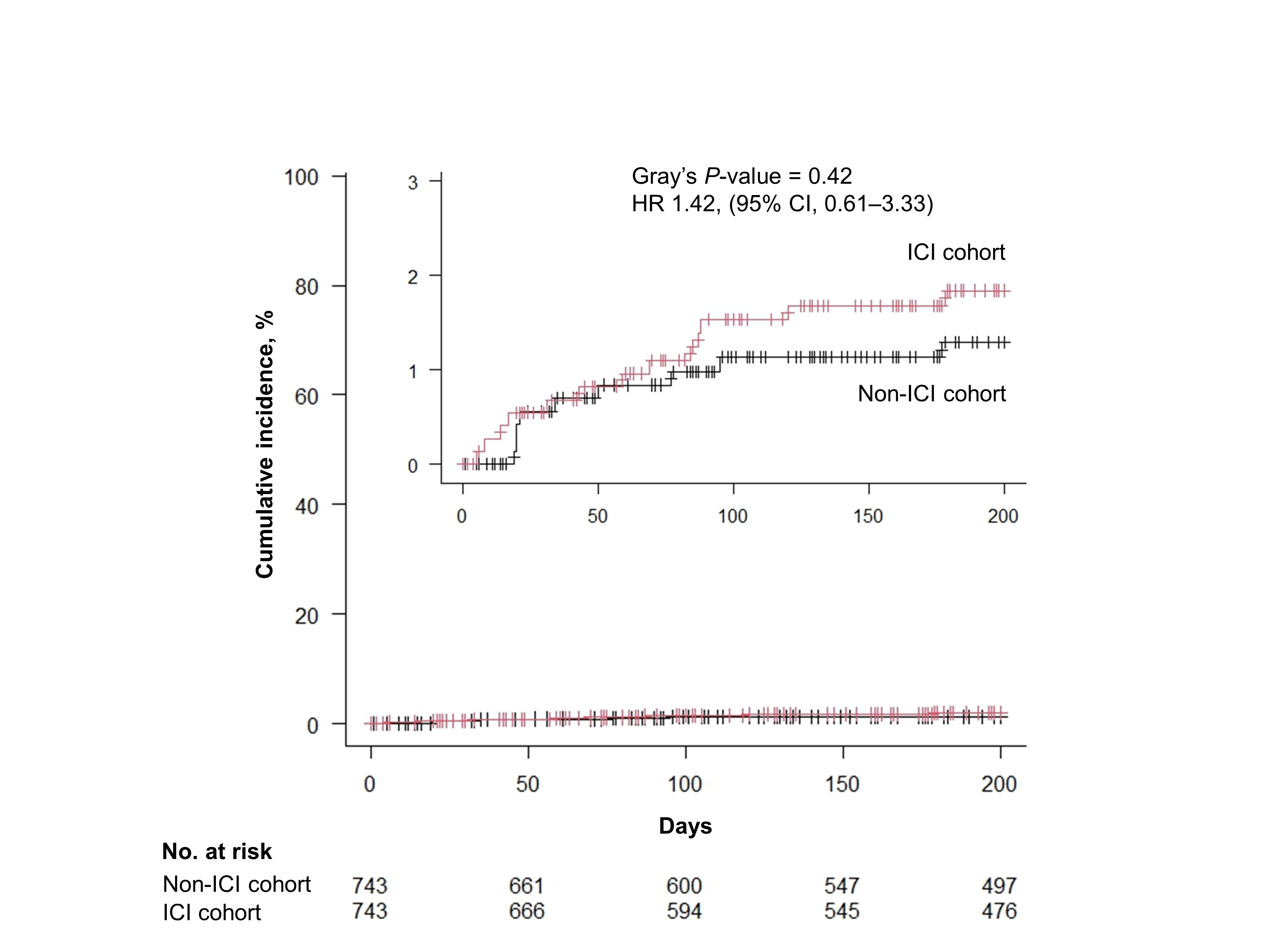

Supplement: oyaf151_suppl_Supplementary_Tables_1-11_Figures_1-8 [file oyaf151_suppl_supplementary_tables_1-11_figures_1-8.zip › Supplementary Figure 7.tif]

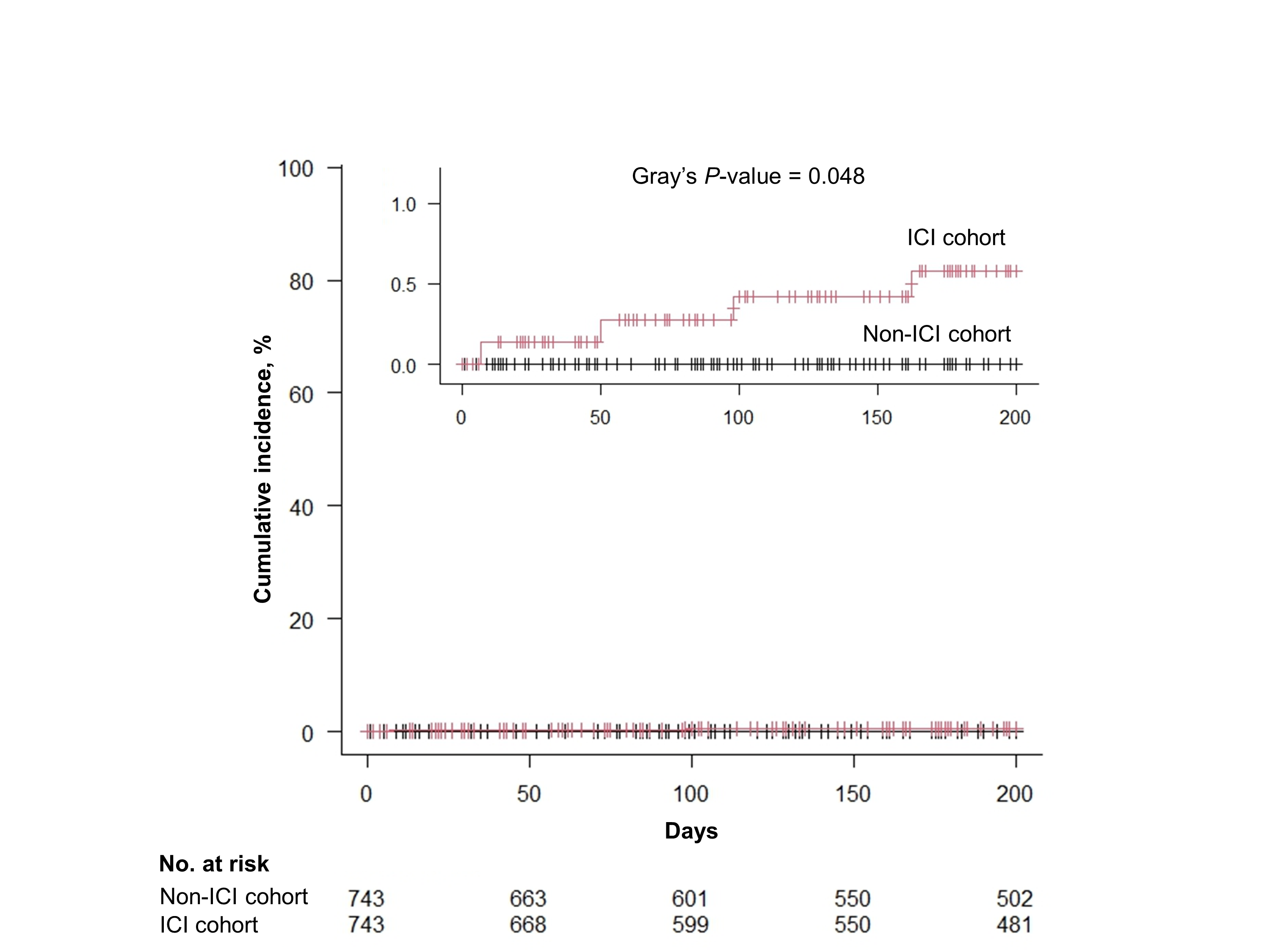

Supplement: oyaf151_suppl_Supplementary_Tables_1-11_Figures_1-8 [file oyaf151_suppl_supplementary_tables_1-11_figures_1-8.zip › Supplementary Figure 4.tif]

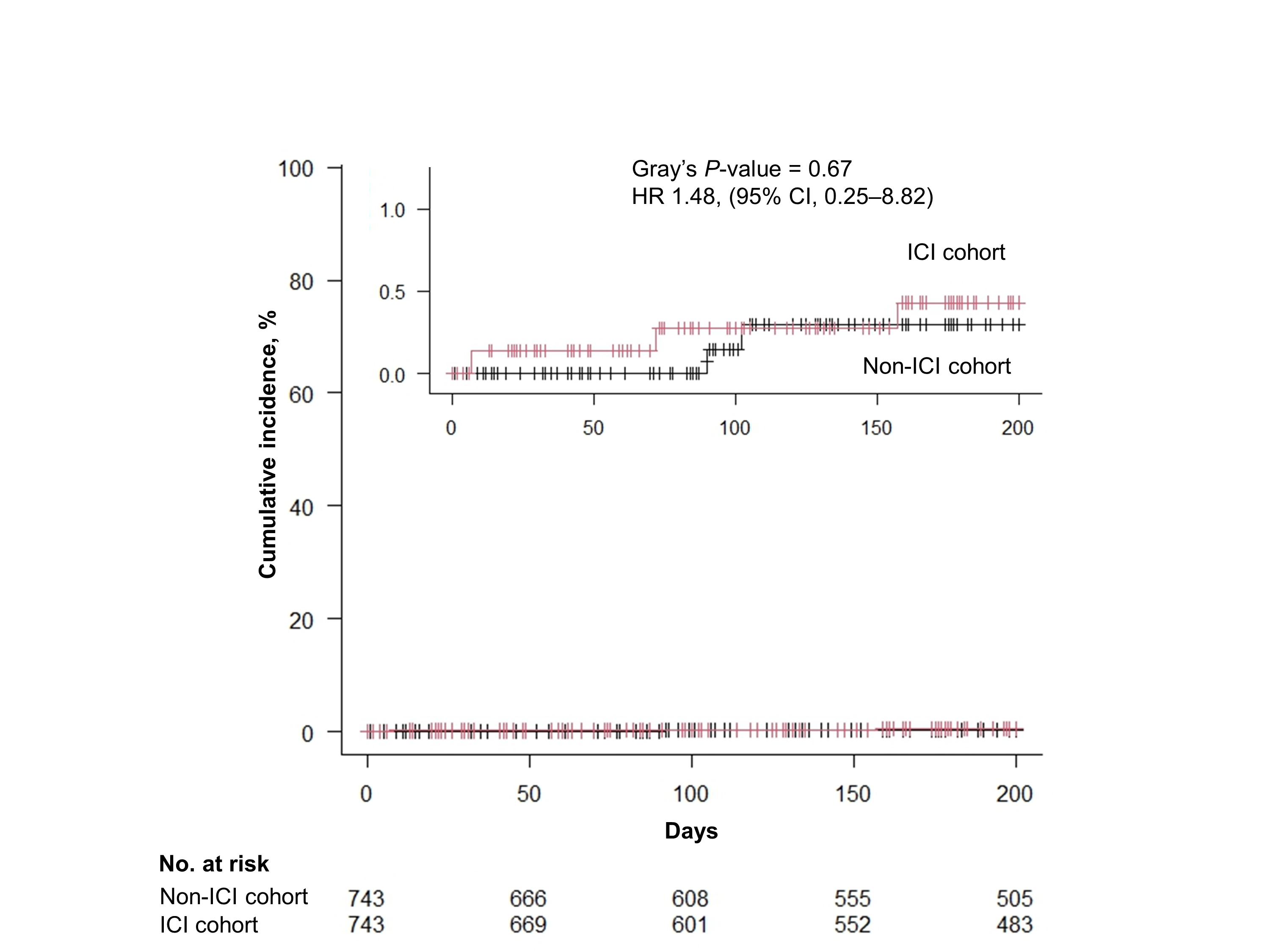

Supplement: oyaf151_suppl_Supplementary_Tables_1-11_Figures_1-8 [file oyaf151_suppl_supplementary_tables_1-11_figures_1-8.zip › Supplementary Figure 8.tif]

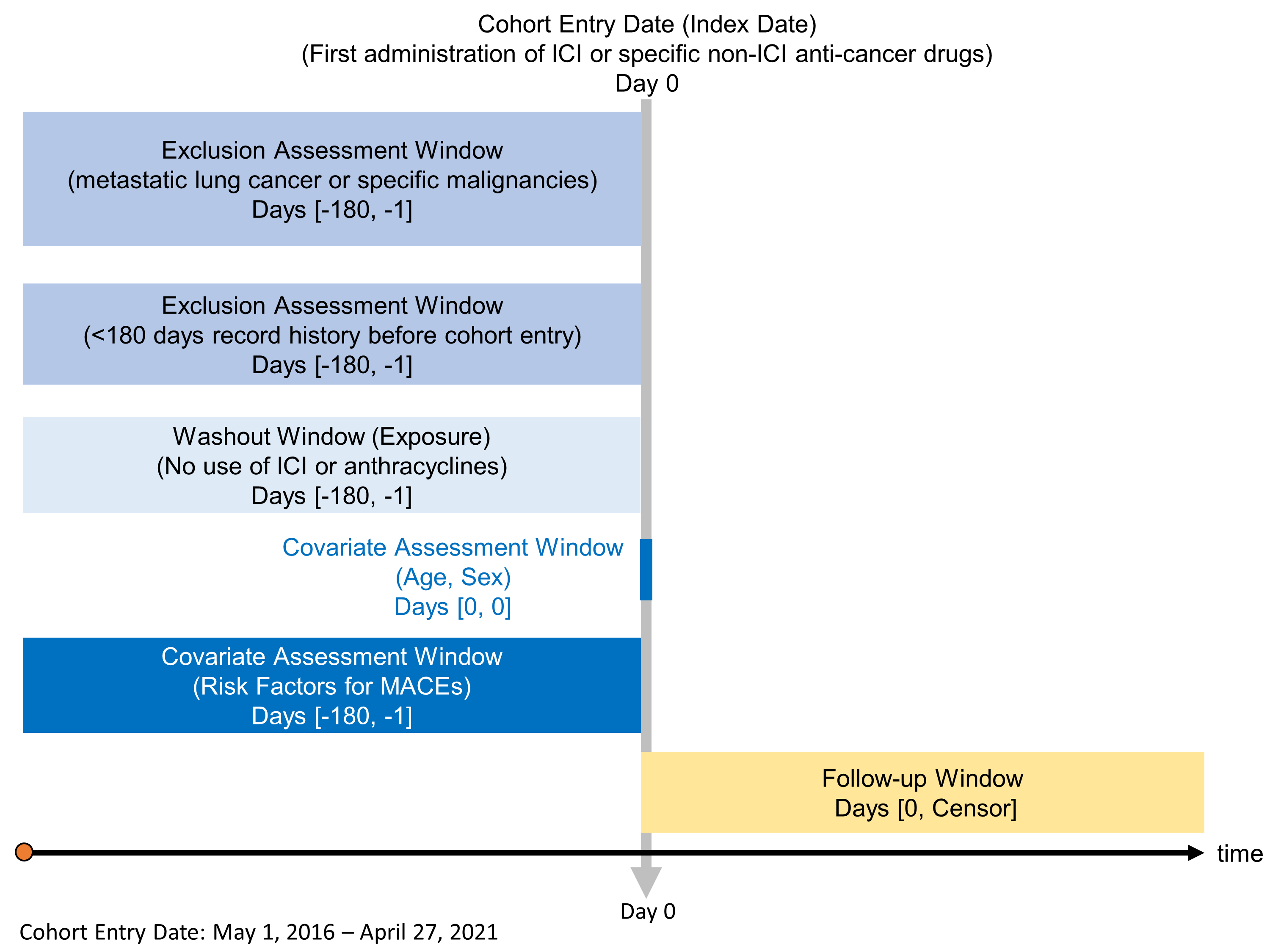

Supplement: oyaf151_suppl_Supplementary_Tables_1-11_Figures_1-8 [file oyaf151_suppl_supplementary_tables_1-11_figures_1-8.zip › Supplementary Figure 1.tif]

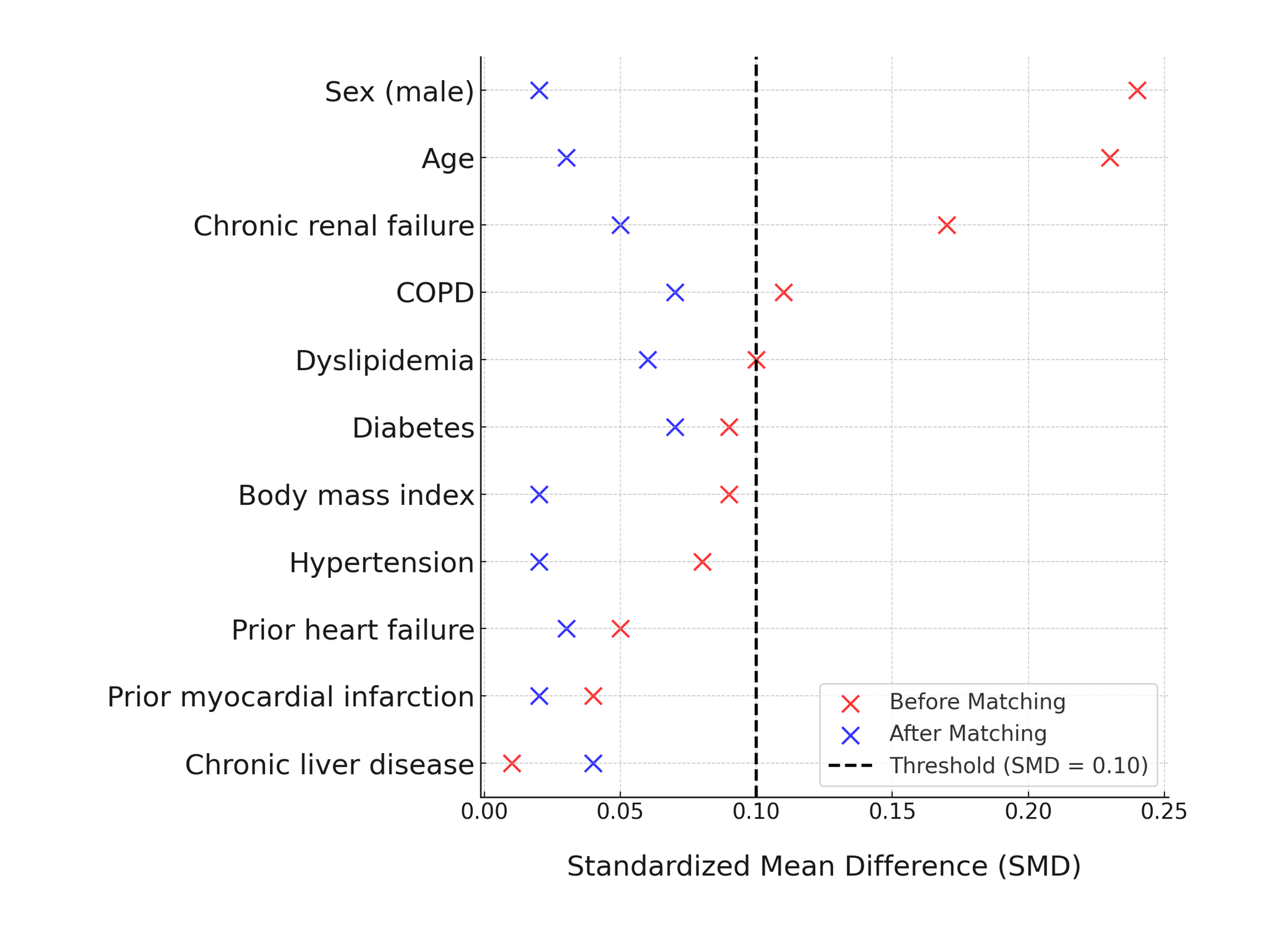

Supplement: oyaf151_suppl_Supplementary_Tables_1-11_Figures_1-8 [file oyaf151_suppl_supplementary_tables_1-11_figures_1-8.zip › Supplementary Figure 2.tif]

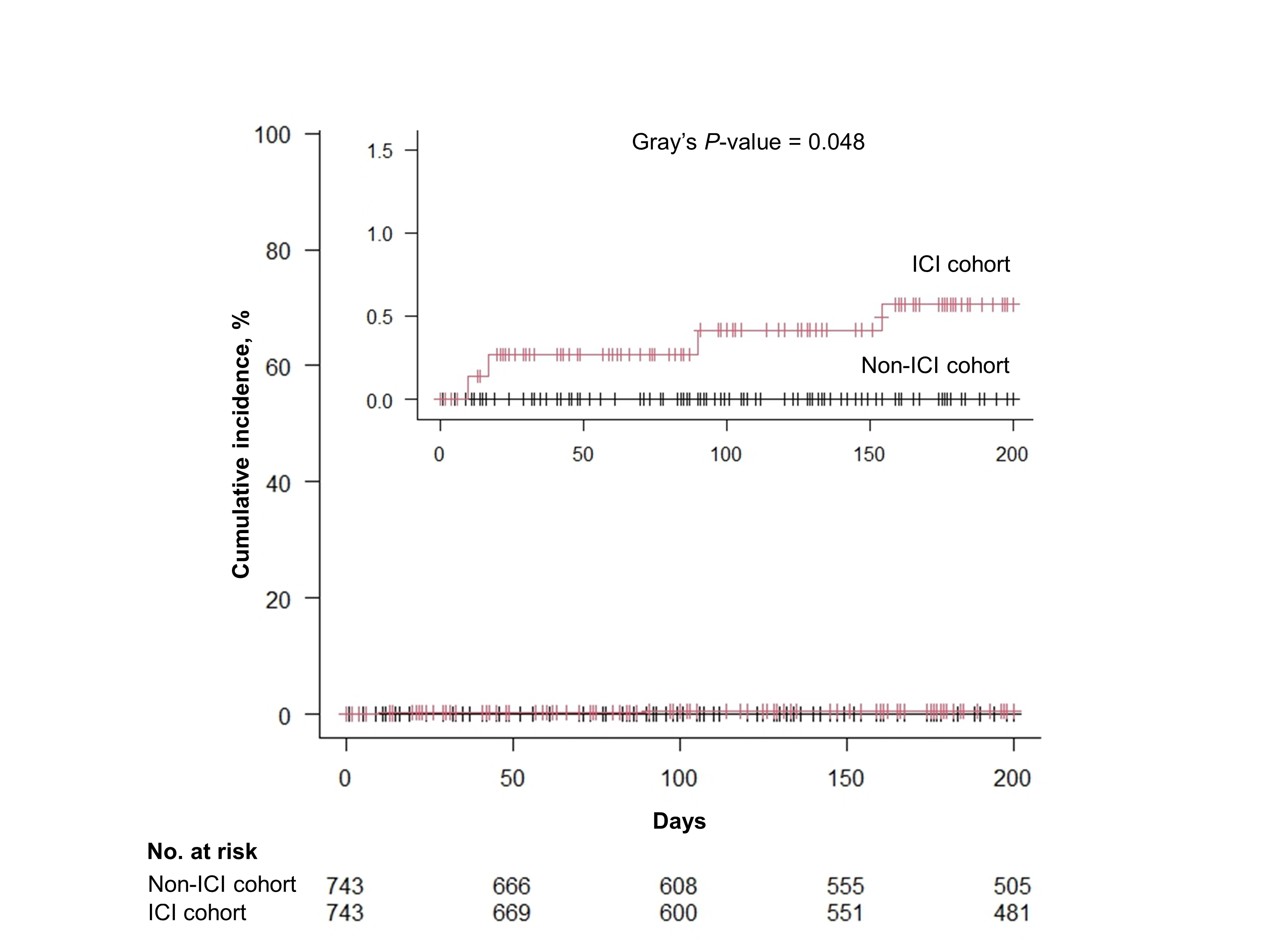

Supplement: oyaf151_suppl_Supplementary_Tables_1-11_Figures_1-8 [file oyaf151_suppl_supplementary_tables_1-11_figures_1-8.zip › Supplementary Figure 3.tif]
